# Navigating the Terrain of Post‐Polypectomy Surveillance: Charting the Complex Landscape of Guidelines and Recurrence Risks

**DOI:** 10.1155/cjgh/2782582

**Published:** 2026-04-27

**Authors:** Xiaojin Tao, Jun Yao

**Affiliations:** ^1^ Department of Gastroenterology, The Affiliated People’s Hospital of Jiangsu University, Zhenjiang, Jiangsu, China, jdfy.cn

**Keywords:** colonoscopy, colorectal adenomas, guidelines, recurrence, surveillance

## Abstract

Colorectal cancer (CRC) remains a leading cause of global cancer‐related morbidity and mortality. The majority of CRC cases arise through two well‐established pathways: the conventional adenoma–carcinoma sequence and the serrated neoplasia pathway. Colonoscopic polypectomy significantly reduces the incidence of CRC, yet postoperative recurrence rates remain substantial, underscoring the critical role of post‐polypectomy colonoscopic surveillance (PPCS). Recent updates to PPCS guidelines by major gastroenterological societies—including the US Multisociety Task Force (USMSTF), European Society of Gastrointestinal Endoscopy (ESGE), British Society of Gastroenterology/Association of Coloproctology of Great Britain and Ireland/Public Health England (BSG/ACPGBI/PHE), and the Asia‐Pacific Task Force—reflect evolving strategies for risk stratification and surveillance intervals. While all guidelines prioritize colonoscopy resources for high‐risk populations and reduce burden for low‐risk individuals, significant variations persist in definitions of high‐risk adenomas (HRA), recommendations for surveillance intervals, and management of specific histological subtypes such as villous architecture. This review comprehensively compares these updated guidelines, highlighting consensus and discordance in clinical recommendations. Furthermore, it synthesizes evidence on multifactorial recurrence risks, encompassing baseline adenoma characteristics, patient‐specific factors, and the quality of endoscopic procedures. Regarding the association with the same risk factor, traditional adenomas and serrated polyps may demonstrate heterogeneity. Understanding these elements is essential for optimizing personalized surveillance strategies and reducing recurrent adenoma burden. Finally, we hope that this review will provide decision‐making support for countries lacking standalone guidelines and help clinicians navigate complex and contradictory guideline recommendations.

## 1. Introduction

According to the latest 2021 report from the International Agency for Research on Cancer (IARC), colorectal cancer (CRC) ranks as the third most common cancer type worldwide and the second leading cause of cancer‐related deaths globally, imposing a heavy economic burden on society [1]. Most CRCs are believed to originate from precancerous polyps, primarily of two pathological types: conventional adenomas and serrated polyps (SPs) [2, 3]. Colonoscopic polypectomy has revolutionized the prevention of CRC by effectively interrupting these pathological processes, thereby reducing CRC incidence and mortality [4]. However, postoperative recurrence rates for polyps can be as high as 20%–50% [5]. Consequently, post‐polypectomy colonoscopic surveillance (PPCS) has become an indispensable component of CRC prevention programs. Its purpose is to detect and remove recurrent or missed polyps at an early stage, preventing their progression to advanced tumors or invasive cancer. The purpose of the PPCS guidelines is to determine the appropriate timing for surveillance after the initial colonoscopy in screening populations, taking into account multiple factors such as CRC risk, colonoscopy burden, and complication risk. In recent years, professional societies worldwide have updated their PPCS guidelines, including the US Multispecialty Task Force (USMSTF) [6], the European Society of Gastrointestinal Endoscopy (ESGE) [7], the British Society of Gastroenterology/Association of Coloproctology of Great Britain and Ireland/Public Health England (BSG/ACPGBI/PHE) [8], and the Asia‐Pacific Task Force [9]. Although these updated guidelines exhibit significant variations in risk stratification criteria and recommended surveillance intervals, they share a consistent trend: all updated guidelines recommend prioritizing colonoscopy resources for high‐risk populations while minimizing the burden of colonoscopy for low‐risk individuals. Understanding these updates and the differences between guidelines is critical for gastroenterologists, endoscopists, and healthcare policymakers. This directly impacts clinical decision‐making, resource allocation, and the development of standardized protocols to minimize both over‐screening and under‐screening. While previous studies have examined differences among various PPCS guidelines, none have addressed the Asia‐Pacific consensus guidelines [10–12]. Notably, the Asia‐Pacific region bears a disproportionately heavy burden of CRC, accounting for the highest number of cases globally and ranking among the highest mortality rates worldwide [9]. Equally important is a comprehensive understanding of the multifactorial nature of adenoma recurrence. Although consensus exists on the carcinogenic pathways, the risk factors contributing to recurrence remain uncertain. The risk of recurrence is influenced by the complex interplay of multiple factors, including adenoma characteristics, patient attributes, and endoscopic procedure. Although traditional adenomas and SPs share many common risk factors for recurrence, there may be heterogeneity in the association between these two precancerous lesions and the same risk factor. In‐depth analysis of these recurrence factors is crucial for optimizing risk stratification models. This review aims to compare the latest PPCS guidelines from major international societies and to analyze risk factors and potential mechanisms associated with adenoma recurrence.

## 2. Comparison of the Latest Guidelines

### 2.1. Main Definitions of Adenomas in the Guidelines

Table 1 summarizes and compares the latest important definitions in the four guidelines.

**TABLE 1:** The latest important definitions of colorectal adenoma in the four guidelines.

Baseline colonoscopy finding	2020 USMSTF/2022 Asia‐pacific consensus	2020 ESGE	2020 BSG/ACPGBI/PHE
1‐2 TA < 10 mm	Low‐risk adenoma 1–2 nonadvanced adenomas^a^ < 10 mm	Polyp not requiring surveillance • 1–4 < 10 mm adenomas with low‐grade dysplasia, irrespective of villous components • Any SP < 10 mm without dysplasia	Premalignant polyp with at least one of the following findings: • SPs (excluding diminutive [1–5 mm] rectal HPs) • Adenomatous polyps

3‐4 TA < 10 mm	High‐risk adenoma with at least one of the following findings: • Advanced neoplasia^b^/advanced adenoma^c^ • ≥ 3 adenomas	Polyp not requiring surveillance	Premalignant polyp

5–10 TA < 10 mm	High‐risk adenoma	Polyp requiring surveillance with at least one of the following findings: • Adenoma ≥ 10 mm • Adenoma with HGD • ≥ 5 adenomas • SP ≥ 10 mm • Any SP with dysplasia	High‐risk findings with at least one of the following findings: • ≥ 2 premalignant polyps including at least 1 advanced colorectal polyp^d^ • ≥ 5 premalignant polyps

> 10 adenomas	High‐risk adenoma	Polyp requiring surveillance	High‐risk findings

Adenoma ≥ 10 mm	AA with at least one of the following findings: • Adenoma ≥ 10 mm • Adenoma with tubulovillous/villous histology • Adenoma with HGD	Polyp requiring surveillance	AA with at least one of the following findings: • Adenoma ≥ 10 mm • Adenoma with HGD

Adenoma with HGD	Advanced adenoma	Polyp requiring surveillance	Advanced adenoma

Adenoma with tubulovillous/villous histology, < 10 mm, LGD	Advanced adenoma	Polyp not requiring surveillance	Premalignant polyp

AA and CRC	Advanced neoplasia	Advanced neoplasia	Advanced neoplasia This term is considered outmoded because it combines two clinically distinct entities and excludes the serrated pathway

1–10 SSL < 10 mm, without dysplasia	^∗^Because evidence regarding the risk of metachronous neoplasia associated with SPs is evolving, we have chosen not to include SSLs and HPs in our definitions of low‐risk adenoma, high‐risk adenoma, and advanced neoplasia, and we will refer to these lesions separately	Polyp not requiring surveillance	Premalignant polyp/high‐risk findings

SP ≥ 10 mm or with dysplasia	^∗^Advanced serrated polyp (SP) with at least one of the following findings: • HP or SSL ≥ 10 mm • SSL with dysplasia • TSA	Polyp requiring surveillance	Advanced SP with at least one of the following findings: • SP ≥ 10 mm • SP with any grade of dysplasia

*Note:* ACPGBI = Association of Coloproctology of Great Britain and Ireland, CRC = colorectal cancer, mm = millimeters.

Abbreviations: AA = Advanced adenoma, BSG = British Society of Gastroenterology, ESGE = European Society of Gastrointestinal Endoscopy, HGD = high‐grade dysplasia, HP = hyperplastic polyp, LGD = low‐grade dysplasia, PHE = Public Health England, SP = serrated polyp, SSL = sessile serrated lesion, TA = tubular adenoma, TSA = traditional serrated adenoma, USMSTF = U.S. Multisociety Task Force.

^∗^Proposed by the USMSTF but not included in the Asia‐Pacific consensus.

^a^Nonadvanced adenomas refer to tubular adenomas < 10 mm and with LGD.

^b^The USMSTF guidelines: high‐risk adenoma includes advanced neoplasia or ≥ 3 adenomas.

^c^The Asia‐pacific Consensus: high‐risk adenoma includes advanced adenoma or ≥ 3 adenomas.

^d^The BSG/ACPGBI/PHE guidelines: advanced colorectal polyp includes AA or advanced serrated polyp.

#### 2.1.1. USMSTF

According to USMSTF guidelines, definitions associated with conventional adenomas are provided, including advanced adenoma (AA), advanced neoplasia, low‐risk adenoma, and high‐risk adenoma. Among these, AA is defined as an adenoma ≥ 10 mm in size, an adenoma containing villous components, or an adenoma with high‐grade dysplasia (HGD). Advanced neoplasia, on the other hand, refers to the combination of AA and CRC. The above definitions do not address the number of adenomas; however, many previous studies have suggested that patients with multiple adenomas (often ≥ 3 in number) have an increased risk of developing synchronous progressive tumors, and the extent of this effect may differ from that of advanced neoplasia [13–15]. Therefore, the USMSTF proposes that the distinction between high‐risk adenoma and advanced neoplasia should not be blurred. Specifically, high‐risk adenomas (HRAs) are defined as advanced adenomas or ≥ 3 adenomas, whereas low‐risk adenomas are defined as 1–2 tubular adenomas < 10 mm with low‐grade dysplasia.

#### 2.1.2. ESGE

Considering that the public may have an overly negative perception of the previous terms, the ESGE guidelines decided to use the term “polyps” instead of “lesions” or “tumors.” Furthermore, in the classification of polyps, the guidelines use the terms “need” or “no need” for surveillance instead of “high risk” and “low risk,” which is very different from the other three guidelines. Patients with adenomas requiring surveillance have one or more of the following characteristics in their baseline colonoscopy: at least 1 adenoma ≥ 10 mm or with HGD or ≥ 5 adenomas. Patients with 1–4 adenomas < 10 mm and without HGD, regardless of villous components, are considered to have polyps that do not need surveillance.

#### 2.1.3. BSG/ACPGBI/PHE

The BSG/ACPGBI/PHE guidelines use the term “premalignant polyp” to encompass both adenomatous and SPs. High‐risk findings are defined as follows: (1) ≥ 2 premalignant polyps (at least one advanced colorectal polyp) or (2) ≥ 5 premalignant polyps. Contrary to the USMSTF guidelines, the BSG/ACPGBI/PHE guidelines do not consider tubulovillous or villous histology as advanced adenomatous polyps.

#### 2.1.4. Asia‐Pacific Consensus

The Asia‐Pacific Consensus aligns with the USMSTF guidelines in defining low‐risk adenomas (one or two tubular adenomas < 10 mm) and advanced adenomas (adenomas > 10 mm, villous histology, HGD). In contrast, the 2022 Asia‐Pacific consensus does not provide a detailed definition of advanced neoplasia (though the Asia‐Pacific working group had defined it as invasive carcinoma or AA in 2011) and defines HRAs as AAs or the presence of ≥ 3 adenomas.

#### 2.1.5. SPs

According to the 2019 World Health Organization recommendations, SPs are classified into four categories: hyperplastic polyps (HPs), sessile serrated lesions (SSLs), traditional serrated adenomas (TSAs), and unclassified serrated adenomas [16]. Notably, “SSL” is used to replace the previous term “sessile serrated adenoma/polyp (SSA/P),” though the 2020 USMSTF guidelines still employ the older terminology. While the 2020 ESGE guidelines mention “SSL” in their evidence section, their recommendations prefer the umbrella term “SPs.” In contrast, the 2020 BSG/ACPGBI/PHE guidelines define this umbrella term as encompassing HPs, SSLs, SSLs with dysplasia (SSLd), TSAs, and mixed polyps. Furthermore, the 2022 Asia‐Pacific consensus not only directly references the WHO terminology update but also points out its significance in emphasizing that such SPs may lack morphological dysplasia or polypoid structural features. Continuing to use the old terminology may cause conceptual confusion.

HPs are generally considered to have low malignant potential, whereas SSLs and TSAs are clearly precancerous lesions [17]. The USMSTF considers individuals with only HP < 10 mm as having had normal colonoscopy. Regarding high‐risk criteria for SPs, the USMSTF and BSG/ACPGBI/PHE guidelines explicitly introduced the term “advanced serrated polyp.” The former defines it as HP or SSL ≥ 10 mm, SSL with dysplasia, or TSA, while the latter defines it as SP ≥ 10 mm or with any grade of dysplasia. However, the ESGE and Asia‐Pacific consensus guidelines do not mention this term. Although the ESGE and Asia‐Pacific consensus guidelines do not explicitly define this term, they both mention similar criteria for managing surveillance.

### 2.2. Main Recommendations for Colorectal Adenomas in the PPCS Guidelines

**FIGURE 1:**
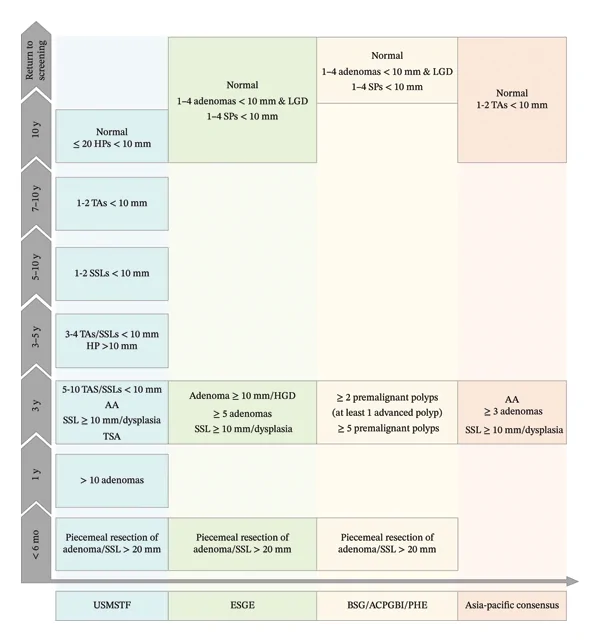
Comparison of main recommendations in the four guidelines. Advanced adenomas (AAs) refer to adenomas ≥ 10 mm or those with HGD or tubulovillous/villous adenomas [6–9]. USMSTF = US Multisociety Task Force, ESGE = European Society of Gastrointestinal Endoscopy, BSG = British Society of Gastroenterology, ACPGBI = Association of Coloproctology of Great Britain and Ireland, PHE = Public Health England, y = years, mo = months, mm = millimeters, HP = hyperplastic polyp, TA = tubular adenoma, SSL = sessile serrated lesion, TSA = traditional serrated adenoma, HGD = high‐grade dysplasia, SP = serrated polyp, LGD = low‐grade dysplasia.

Table 2 and Figure 1 summarize and compare the latest surveillance recommendations for colorectal adenomas in the four guidelines.

**TABLE 2:** Comparison of main recommendations for conventional adenomas in the four guidelines.

Baseline colonoscopy finding	2020 USMSTF	2020 ESGE	2020 BSG/ACPGBI/PHE	2022 Asia‐pacific consensus
Normal	10 years	Return to screening	No surveillance/return to screening when invited^b^	Return to screening/10 years^e^
Conventional adenomas				
Nonadvanced adenomas (No. of TA < 10 mm)				
1‐2	7–10 years	Return to screening/10 years^a^	No surveillance/return to screening when invited^b^	Return to screening/10 years^e^
3‐4	3–5 years	Return to screening/10 years^a^	No surveillance/return to screening when invited^b^	3 years
5–10	3 years	3 years	3 years	3 years
Advanced adenomas				
≥ 10 mm or with HGD	3 years	3 years	3 years^c^	3 years
with tubulovillous/villous histology, < 10 mm, LGD	3 years	Return to screening/10 years^a^	No surveillance/return to screening when invited^b^	3 years
> 10 adenomas	1 year and genetic counseling	Genetic counseling	Referred to BSG hereditary CRC guidelines (1‐2 years)	3 years
Piecemeal resection of adenoma > 20 mm	6 months	3–6 months	2–6 months^d^	—
Serrated polyps (SPs)				
≤ 20 HPs, < 10 mm				
in rectum or sigmoid colon	10 years	—	—	—
proximal to sigmoid colon	10 years	—	—	—
No. of SSL < 10 mm, without dysplasia				
1‐2	5–10 years	Return to screening/10 years^a^	No surveillance/return to screening when invited^b^	—
3‐4	3–5 years	Return to screening/10 years^a^	No surveillance/return to screening when invited^b^	—
5–10	3 years	—	3 years	—
Advanced SP				
HP ≥ 10 mm	3–5 years	3 years	3 years^c^	—
SSL ≥ 10 mm or with dysplasia	3 years	3 years	3 years^c^	3 years
TSA ≥ 10 mm or with dysplasia	3 years	3 years	3 years^c^	—
SPS	—	—	Referred to BSG hereditary CRC guidelines (1–2 years)	—
Piecemeal resection of SSL > 20 mm	6 months	3–6 months	2–6 months^d^	—

*Note:* ACPGBI = Association of Coloproctology of Great Britain and Ireland, CRC = colorectal cancer, mm = millimeters.

Abbreviations: BSG = British Society of Gastroenterology, ESGE = European Society of Gastrointestinal Endoscopy, HGD = high‐grade dysplasia, HP = hyperplastic polyps, LGD = low‐grade dysplasia, PHE = Public Health England, SP = serrated polyp, SPS = serrated polyposis syndrome, SSL = sessile serrated lesion, TA = tubular adenoma, TSA = traditional serrated adenoma, USMSTF = U.S. Multi‐Society Task Force.

^a^The ESGE recommends repeating colonoscopy after 10 years if participation in an organized screening program is not feasible.

^b^The BSG/ACPGBI/PHE recommends no surveillance but advises participation in the national bowel screening program when invited.

^c^The BSG/ACPGBI/PHE recommends surveillance after 3 years for patients with ≥ 2 precancerous polyps.

^d^The BSG/ACPGBI/PHE recommends surveillance of the site at 18 months after initial resection to monitor for late recurrence.

^e^The Asia‐Pacific consensus recommends extending surveillance intervals to 10 years only when based on a high‐quality baseline colonoscopy.

#### 2.2.1. 1–4 Tubular Adenomas < 10 mm

Compared with previous guidelines, the updated recommendations in these four guidelines have extended surveillance intervals for tubular adenomas < 10 mm. However, there are slight differences in the exact intervals recommended by each guideline. The USMSTF recommends that individuals with baseline nonadvanced adenomas (NAAs) of 1–2 and 3–4 repeat colonoscopy at 7–10 years and 3–5 years, respectively. However, in its 2012 guidelines, the USMSTF classified these two groups as high‐risk populations, recommending follow‐up intervals of 5–10 years and 3 years, respectively [18]. This shift to longer surveillance intervals is based on new research confirming that patients with baseline low‐risk adenomas do not have an increased risk of developing synchronous advanced tumors and CRC during follow‐up [19, 20].

For individuals with 1–4 adenomas < 10 mm with LGD, contrary to the USMSTF, the ESGE and BSG/ACPGBI/PHE do not recommend regular endoscopic surveillance. ESGE recommends that patients with these adenomas return to their local screening programs (primarily via fecal immunochemical testing [FIT]), as their long‐term risk of CRC incidence and mortality is lower than or similar to that of the general population. For countries without organized screening programs, ESGE suggests colonoscopy after 10 years as an alternative option. Similarly, BSG/ACPGBI/PHE emphasizes that patients should participate in national CRC screening programs (such as FIT) when invited, rather than advocating colonoscopy surveillance. This stance contrasts with their previous guidelines: ESGE in 2013 recommended a 3‐year surveillance interval for individuals with ≥ 3 adenomas, and BSG/ACPGBI/PHE in 2010 also recommended a 3‐year surveillance cycle for patients with 3–4 small adenomas [21, 22]. Regarding surveillance recommendations for individuals with such adenomas, the updated trends of these two guidelines are consistent, both leaning toward extending the surveillance interval.

Although the 2022 Asia‐Pacific consensus shares a conservative stance with the USMSTF regarding extending surveillance intervals for patients with 1–4 tubular adenomas < 10 mm, they diverge in their specific recommendations. The 2022 Asia‐Pacific Consensus recommends that individuals with baseline NAAs of 1–2 undergo repeat colonoscopy after 10 years or return to routine screening, compared with 5–10 years in the 2015 guidelines. For individuals with baseline NAAs of 3–4, both the 2022 and 2015 Asia‐Pacific Consensus recommend a surveillance interval of 3 years [23].

#### 2.2.2. Adenomas ≥ 10 mm or With High‐Grade Dysplasia

All four guidelines recommend a 3‐year surveillance interval for individuals with baseline colonoscopy findings of adenomas ≥ 10 mm or HGD. However, it is noteworthy that the BSG/ACPGBI/PHE guidelines provide quantitative criteria: the 3‐year surveillance interval is recommended only for patients with two or more precancerous polyps (i.e., those with high‐risk findings), but not for those with only one precancerous polyp.

#### 2.2.3. Adenomas With Tubulovillous/Villous Histology

For patients with adenomas < 10 mm containing villous histologic components, significant disagreement exists among guidelines. The USMSTF and Asia‐Pacific consensus recommend a 3‐year surveillance interval for such patients, while ESGE and BSG/ACPGBI/PHE do not recommend surveillance colonoscopy and advise participation in the national bowel screening program when invited. The shorter surveillance intervals recommended by the USMSTF and Asia‐Pacific Consensus stem from evidence emerging as the previous guidelines indicate an association between villous histology and increased risk of developing metachronous advanced colorectal neoplasia (ACRN) during follow‐up [13, 14]. However, recent studies suggest no significant association between villous histology and long‐term CRC incidence or mortality risk [24, 25]. Second, there is high interobserver variability among pathologists in assessing villous structures [26]. Finally, the coexistence of villous histology is uncommon in patients with adenomas < 10 mm without HGD [27]. Therefore, the ESGE and BSG/ACPGBI/PHE consider the inclusion of villous components to be unjustified and an unnecessary increase in surveillance burden.

#### 2.2.4. Number of Adenomas

While all four guidelines agree on the importance of surveillance for multiple adenomas, the number of adenomas remains one of the main points of contention among them regarding recommended surveillance intervals. As mentioned above, recommendations for surveillance intervals in individuals with 1–4 adenomas < 10 mm are inconsistent. While all four guidelines recommend repeat colonoscopy screening after 3 years for patients with 5–10 adenomas, their recommendations diverge for patients with > 10 adenomas. The Asia‐Pacific consensus recommends a 3‐year follow‐up colonoscopy for patients with > 10 adenomas, while the USMSTF shortens the surveillance interval to 1 year and additionally proposes genetic counseling. In contrast, the ESGE recommendation includes only genetic counseling. Furthermore, the BSG/ACPGBI/PHE references the BSG hereditary CRC guidelines to guide management of patients with a baseline of > 10 adenomas.

#### 2.2.5. Piecemeal Resection of Adenoma > 20 mm

Compared with en bloc resection, piecemeal resection for colorectal adenomas is associated with significantly higher rates of incomplete resection (as determined by immediate post‐resection biopsy) and recurrence risk. However, after adjusting for adenoma size and histology, piecemeal resection was found not to be an independent predictor of incomplete resection [28, 29]. Furthermore, current evidence regarding follow‐up after piecemeal resection primarily stems from studies involving adenomas > 20 mm [30, 31]. Therefore, three guidelines recommend more intensive surveillance for patients with piecemeal resection of adenomas > 20 mm. The USMSTF advises them to repeat colonoscopy after 6 months, while the ESGE and BSG/ACPGBI/PHE prefer shorter surveillance intervals of 3–6 months and 2–6 months, respectively. In contrast to the above, the Asia‐Pacific consensus does not specifically address surveillance for this patient group, instead treating them as AAs with a 3‐year surveillance interval.

#### 2.2.6. SPs

Surveillance recommendations for SPs show considerable variation, reflecting the limited evidence base and evolving understanding of their natural history (Table 2). Due to a lack of research on the risk of metachronous ACRN associated with SPs, some guidelines do not provide detailed recommendations on surveillance for patients, particularly those with small SPs without dysplasia. For example, among the four guidelines, only USMSTF provides detailed recommendations for < 20 HPs (all < 10 mm)—a 10‐year follow‐up interval. In addition, for SSLs < 10 mm without dysplasia, only the USMSTF differentiates based on lesion count and provides detailed recommendations: patients with 1‐2, 3‐4, or 5–10 lesions are recommended to repeat colonoscopy in 5–10 years, 3–5 years, or 3 years, respectively. Although these are weak recommendations based on very low‐quality evidence, they are helpful for clinicians. On the other hand, the ESGE classifies any SP < 10 mm without dysplasia as a “Polyp not requiring surveillance.” While the ESGE shares similarities with the BSG/ACPGBI/PHE guidelines, the latter considers ≥ 5 premalignant polyps as high‐risk findings warranting a 3‐year surveillance interval and recommends considering serrated polyposis syndrome (SPS) when ≥ 20 SPs are present (see BSG hereditary CRC guidelines). For high‐risk SPs (based on respective high‐risk criteria), all four guidelines reach consensus on recommending a 3‐year surveillance interval. Notably, for large HPs (≥ 10 mm), the USMSTF permits 3‐ to 5‐year follow‐up intervals, considering pathologist consistency in distinguishing HP from SSL, complete resection, or satisfactory bowel preparation. For single advanced SP, BSG/ACPGBI/PHE recommends no surveillance, while repeat colonoscopy in 3 years is recommended only when ≥ 2 precancerous polyps are present (with ≥ 1 lesion having advanced features). Compared to the other three guidelines, the Asia‐Pacific Consensus Guidelines only provide recommendations for SSLs > 10 mm or with dysplasia: a colonoscopy every 3 years is advised.

#### 2.2.7. Age to Stop Surveillance

The recommended age for stopping colonoscopy surveillance after adenoma resection varies slightly across guidelines. The USMSTF states that further research is needed to determine whether the benefits of colonoscopy surveillance for potential CRC prevention and early detection outweigh the risks of the procedure itself in patients over 75 years old or with multiple comorbidities. In contrast, the BSG/ACPGBI/PHE offers clearer recommendations: in general, routine colonoscopy surveillance should be stopped in individuals over 75 years old or with an expected lifespan < 10 years. Similarly, the ESGE recommends stopping colonoscopy surveillance at age 80 or earlier if life expectancy is limited by comorbidities. The Asia‐Pacific Consensus emphasizes that clinicians should not rely solely on age to limit screening but should consider the patient’s overall health status, individual preferences, and prior screening history. However, if the most recent colonoscopy was negative, the Asia‐Pacific Consensus suggests stopping surveillance at age 85.

## 3. Analysis of Recurrence Factors

**FIGURE 2:**
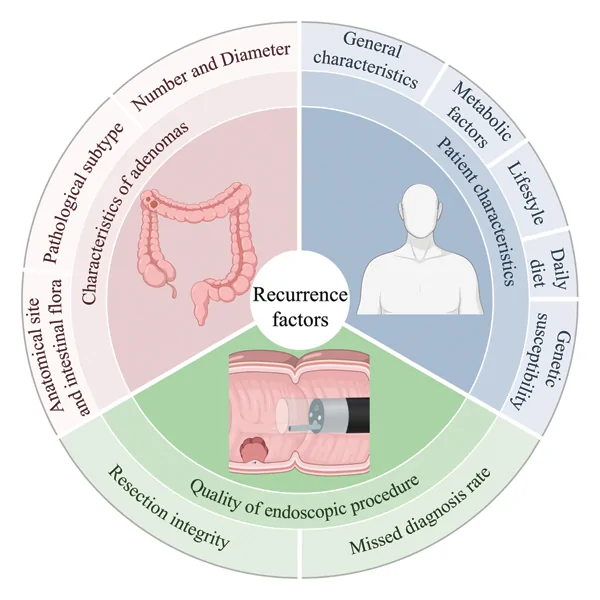
Multiple risks of adenoma recurrence after polypectomy.

The risk of colorectal adenoma recurrence following polypectomy is multifactorial, influenced by a complex interplay of lesion characteristics, patient‐specific factors, and procedural quality (Table 3; Figure 2). Previous studies rarely distinguish between SPs and conventional adenomas when evaluating recurrence risk factors. Given that literature specifically examining recurrence of SPs remains relatively scarce and surveillance recommendations are relatively weak, we primarily reviewed risk factors for conventional adenoma recurrence while contrasting them with existing evidence related to SPs (Table 4), hoping to help deepen understanding of the serrated pathway and improve surveillance guidelines.

**Table 3:** Risk factors for colorectal adenoma recurrence following polypectomy.

Category	Risk factor	Specific features	Proposed mechanism	Ref.
Adenoma characteristics	Number	≥ 3 (whether overall or advanced adenomas)	Missed diagnosis and incomplete resection	[32–35]
	Diameter	≥ 10 mm/> 20 mm	Faster growth rate; higher likelihood of residual lesions	[18, 37]
	Adenoma bulk	Sum of all baseline adenoma diameters ≥ 1 cm	Cumulative effect of multiple lesions	[38]
	Villous structures	Villous adenoma (> 75% villous)	Rapid cell division; high proportion of atypical hyperplasia	[44–46]
	Dysplasia grade	High‐grade dysplasia	Structural/cytological abnormalities; higher malignant potential	[30, 46–48]
	Anatomical site	Proximal colon	Microenvironmental differences; technical difficulty; incomplete resection	[55–59]
	Intestinal flora	Changes in the abundance of certain bacterial genera (e.g., *Haemophilus* and *Fusobacterium nucleatum*)	Microbial dysbiosis or amino acid alterations	[61, 62, 69]

General patient characteristics	Age	Each additional year	Age‐related immunosenescence; cumulative genomic instability; prolonged exposure to environmental carcinogens	[70–73]
	Sex	Male	Hormonal differences; behavioral factors	[37, 77, 78]
	Race/ethnicity	Non‐Hispanic whites	Genetic, environmental, and cultural factors	[37, 84]

Metabolic and lifestyle factors	Glucose metabolism	Elevated fasting glucose/diabetes	Increased sensitivity to oxidative stress; affects IGF signaling	[87, 89–91]
	Smoking	History ≥ 35 years or ≥ 30 cigarettes/day	Carcinogen‐induced oxidative stress and DNA damage	[92–95]
	Alcohol consumption	≥ 2 times/day	Acetaldehyde and oxygen‐free radicals produced; inflammatory cytokine changes	[99–101]
	Obesity	Higher BMI	Increased insulin and IGF‐I levels	[102, 103]
	Habit	Sedentary behavior	Low physical activity	[104]
	Physical activity	≥ 1 h/week exercise (protective)	Long‐term aerobic exercise reduces resting heart rate	[105–107]
	Dyslipidemia	Elevated levels of apolipoprotein B and triglycerides	Visceral fat deposition; inflammatory cytokine changes; tumor stem cells rich in lipid droplets	[35, 108–111]

Dietary patterns and nutritional factors	Dietary patterns	Western dietary pattern/Mediterranean dietary pattern (protective)	Former: high intake of red/processed meat, refined carbohydrates, added sugars, saturated fat; Latter: rich in fruits, vegetables, legumes, whole grains, fish, and olive oil	[112, 115]
	Red/processed meat	Fried, processed, well‐done/very well‐done	Increase the production of mutagens	[120, 121]
	Dietary fiber (protective)	Proximal adenomas and specific fiber subtypes (cereals, fruits, and vegetables)	Diluting fecal carcinogens, shortening colonic transit time, and promoting butyrate production	[124, 125]
	Total fat/saturated fat	High intake	Increases bile acid secretion	[128, 130]
	Added sugars	Excessive intake	Increases postprandial insulin secretion	[89, 131, 132]
	Micronutrients	Calcium (protective)/Vitamin D (protective)	Calcium binds to the bile/lipids; Vitamin D regulates cell proliferation, differentiation, and apoptosis via VDR	[133–137]
	Green tea	Green tea extract (protective)	EGCG inhibits COX‐2 and IGF‐1 receptor; induces apoptosis	[142–145]

Genetic susceptibility	*IL10*/*PTGS2* (COX‐2)/*GC*	*IL10*‐1082 G> A (A allele) & NSAIDs; *PTGS2* rs5277 CC, rs4648310 AG, rs4648319; *GC* rs4588 CC & supplementation (Vit D3/calcium)	Inflammatory signaling pathway	[139, 148–150]
	*APC*/*ODC1*/*TCF7L2*	*APC* TA haplotype (codons 486 & 1822); *ODC1* rs2302615 G316A (A allele); *TCF7L2* rs7903146 T allele	Wnt/β‐catenin pathway	[151–154]
	*MLH1*/*MSH6*/*MSH6*/*EXO1*	*MLH1* rs1800734 ‐93G > A (AA); *MSH6* rs1042821 Gly39Glu; *MSH3* rs26279 Ala1045Thr (G allele); *EXO1* rs9350 Ser/Phe	DNA repair pathway	[156, 157, 159, 160]
	*IRS1*/*IGF1*/*IGF1R*/*PIK3R1*/*INSR*	*IRS1* G972R (R allele); *IGF1* non‐192 allele; *IGF1R* rs7166348 A allele & *PIK3R1* rs1823023 AA; *IGF1R* A & *PIK3R1* any G & *INSR* AA (protective)	Insulin‐IGF signaling pathway	[161, 163]

Procedural quality	Resection integrity	EMR (piecemeal and en bloc)/ESD/UEMR	Differences in surgical methods and operator skills	[29, 163–166]
	Missed diagnosis rate	Small, multiple, or right‐sided polyps; inadequate withdrawal time or bowel preparation	Neglected misdiagnosis rate; operator skills; insufficient inspection, poor visibility	[18, 54, 167–170, 176, 177]

*Note:* COX‐2 = cyclooxygenase‐2; EGCG =  (−)‐epigallocatechin‐3‐gallate; GI = gastrointestinal; NSAIDs = nonsteroidal anti‐inflammatory drugs.

Abbreviations: BMI = body mass index, EMR = endoscopic mucosal resection, ESD = endoscopic submucosal dissection, GTE = green tea extract, IGF = insulin‐like growth factor, NF‐κB = nuclear factor‐kappa B, UEMR = underwater endoscopic mucosal resection, VDR = vitamin D receptor.

**TABLE 4:** Current evidence regarding the recurrence risk of serrated polyps versus conventional adenomas.

Category	Specific features	Serrated polyps	Conventional adenomas
Lesion characteristics	Number	SPS	≥ 3
	Diameter	≥ 10 mm	≥ 10 mm
	Subtype	SSL or TSA	Villous adenoma
	Dysplasia grade	Any grade	High grade
	Anatomical site	Proximal colon	Proximal colon
	Intestinal flora (*F. nucleatum*)	Increase risk	Increase risk

Nonmodifiable risk factors	Age	Stable prevalence with increasing age	Increasing prevalence with age
	Sex	Controversial	Male
	Race/ethnicity	White race (vs African Americans and Hispanics)	Non‐Hispanic whites (vs non‐Hispanic Blacks and Asian/Pacific Islander)
	Genetic susceptibility (*MLH1* rs1800734)	May increase risk (with BRAF V600E somatic mutation)	May increase risk

Modifiable risk factors	Metabolic syndrome	Increase risk	Increase risk
	Smoking	Strongest association; especially for distal lesions	Weaker association
	Alcohol consumption	Weaker and less consistent association	More consistent and stronger association
	Sedentary behavior	Increase risk	Increase risk
	Red meat	Stronger association	Weaker association
	High total fat intake	Controversial	Increase risk

Protective factors	Dietary fiber	May increase risk	Controversial
	Calcium and vitamin D	Controversial	Affected by genetic susceptibility

Omission risk	Morphology	Cryptic morphology	Small, multiple
	Pathological diagnosis	High interobserver variability	More consistent
	Incomplete resection rates	Higher	Lower

Abbreviations: SPS = serrated polyposis syndrome, SSL = sessile serrated lesion, TSA = traditional serrated adenoma.

### 3.1. Characteristics of Adenomas

#### 3.1.1. Number and Diameter

The hypothesis that the number of colorectal adenomas at baseline colonoscopy is closely related to the risk of postoperative recurrence is widely accepted [32–35]. A long‐term postoperative follow‐up study has shown that having three or more adenomas, whether overall or advanced adenomas, is a characteristic of a higher risk of future recurrence for patients. This may be related to missed diagnosis and incomplete resection. Undiscovered or residual adenomas progress slowly until they can be detected during surveillance colonoscopy [34].

Meta‐analysis of surveillance colonoscopy data revealed that patients with ≥ 3 adenomas at baseline demonstrated a pooled relative risk of 1.64 for adenoma recurrence, while adenomas ≥ 10 mm showed a relative risk of 1.66 [36]. The latest follow‐up study indicated that adenomas ≥ 1 cm show an increased risk of recurrence, albeit to a lesser extent than histopathologic features [37]. Interestingly, Anderson et al. did not consider advanced features and developed a new indicator, namely, adenoma bulk, which is the sum of all baseline adenoma diameters. They found that patients with an adenoma bulk ≥ 1 cm had a higher risk of progressive adenoma recurrence [38]. The larger the diameter of colorectal adenomas, the faster they grow and the more likely they are to leave residual lesions, which may lead to postoperative recurrence [18]. Furthermore, evidence indicates that the relationship between size and recurrence risk appears nonlinear, with a particularly steep increase observed for adenomas exceeding 20 mm, where incomplete resection rates rise dramatically despite advanced endoscopic techniques [39, 40]. Therefore, it is essential to focus on surveillance of large adenomas.

Regarding SPs, size similarly serves as a critical determinant of recurrence risk. Large cohort studies have shown that patients with baseline SSLs ≥ 10 mm have a two to threefold higher risk of developing metachronous advanced lesion compared with those with only small HPs [41, 42]. When multiple SPs are identified, clinicians should consider the possibility of SPS, which represents a high‐risk phenotype along the serrated pathway. The latest definition criteria for SPS are: ≥ 5 SPs proximal to the rectum (all ≥ 5 mm in size) with ≥ 2 measuring ≥ 10 mm, or > 20 SPs of any size distributed throughout the colon with ≥ 5 proximal to the rectum [16]. A recent prospective 5‐year international cohort study revealed that the cumulative incidence of advanced tumors in SPS patients reached 44% over a 5‐year follow‐up period [43].

#### 3.1.2. Pathological Subtype

As is well known, adenomatous polyps can be classified into tubular (≤ 25%), tubulovillous (26%–75%), and villous (> 75%) adenomas based on the proportion of villous structures. Tubular adenomas are the most common type and carry the lowest risk of metachronous adenoma recurrence and CRC compared with other histologic types. The type most prone to malignant transformation and recurrence is the villous adenoma [44–46]. A meta‐analysis comprising 55 studies with 936,540 patients demonstrated that villous adenomas were associated with a 1.75‐fold increased risk of metachronous CRC compared with tubular adenomas (95% CI, 1.33–2.31) [40]. This association may be explained by the biological characteristics of villous tissue, including increased proliferative activity and a higher degree of cytologic atypia.

There is a consensus that advanced dysplasia is significantly associated with the risk of metachronous advanced neoplasia [34, 40, 46]. The pathological characteristics of HGD are structural abnormalities and/or cytological abnormalities [47]. It is well known that dysplasia can predict the risk of progression to cancer [30, 48]. This may explain why HGD is associated with a high recurrence rate. In a retrospective analysis of 746 patients with advanced colorectal adenomas, Facciorusso et al. reported that the recurrence rate increased substantially with HGD, reaching 57.9% in patients with HGD > 15 mm compared with only 4.2% in those with a single adenoma ≤ 15 mm without HGD [46]. It is worth noting that in patients with HGD at baseline colonoscopy, the risk of adenoma recurrence depends on the diameter. The larger the diameter, the larger the area of HGD, leading to a higher risk of recurrence [46]. Therefore, the adenoma pathological pattern, atypical dysplasia, and diameter should be considered as a complication. These findings align with a systematic review and meta‐analysis investigating risk factors for metachronous CRC and advanced neoplasia, which identified HGD as a significant predictor of adverse outcomes during surveillance [49].

Within the serrated pathway, pathological subtype is equally pivotal. HPs (especially those < 10 mm in the distal colon) are generally considered benign with an extremely low recurrence risk. Conversely, the recurrence rate for SSLs (particularly those ≥ 10 mm, in proximal locations, or with dysplasia) is markedly elevated, comparable to or even exceeding that of conventional adenomas [50]. SSLd represent a high‐risk scenario, capable of rapidly progressing to CRC within a short timeframe, even when exhibiting low‐grade dysplasia [51]. Although TSA is rare, the risk of developing high‐risk metachronous adenomas after resection is significantly higher than with no polyps, even exceeding that of conventional adenomas (47.3% vs. 32.0%) [52, 53]. Notably, a meta‐analysis encompassing 11 studies (*n* = 1,079,315) demonstrated that synchronous SPs with HRAs significantly amplified the risk of metachronous ACRN compared to HRAs alone (OR: 1.64, 95% CI: 1.21–2.24), particularly synchronous SSLs with HRAs (OR: 3.10, 95% CI: 1.92–4.99) [54].

#### 3.1.3. Anatomical Site and Intestinal Flora

The recurrence of colorectal adenomas is related to their anatomical location [55, 56]. Adenomas are more likely to be missed or recur in the proximal colon [57]. Compared with patients whose lesions are confined to the distal colon (i.e., the colon distal to the splenic flexure), patients whose lesions are confined to the proximal colon (i.e., the colon proximal to but not including the splenic flexure) have a higher risk of developing metachronous adenomas. Furthermore, patients with adenomas located only in the proximal colon at baseline are more likely to experience recurrence of adenomas on the same side [58]. This may be related to differences in the microenvironment or the difficulty of colonoscopy in the right half of the colon, leading to incomplete resection [59]. This anatomical predisposition similarly applies to the serrated pathway, where SSLs not only predominantly occur in the proximal colon but also exhibit significantly higher metachronous advanced neoplasia risk in proximal SSLs compared to distal SSLs (HR: 9.3; 95% CI: 5.4–15.9) [41, 60].

A study on colonic microbial abundance used a mucosal classifier to accurately identify adenoma formers (i.e., patients who develop adenomas), regardless of whether the mucosal tissue was adjacent to or distant from the adenoma. They also found that the abundance of *Haemophilus* species, which is associated with increased adenoma burden, was relatively increased in the proximal colon, which may be related to the high incidence of adenomas in the proximal colon [61]. The main microbial community structure of adenoma patients, both before and after surgery, is rarely affected; however, changes in the abundance of certain bacterial genera may predict potential recurrence [62]. Along the adenoma–carcinoma sequence, metagenomic and 16S rRNA studies have consistently demonstrated that gut microbial dysbiosis is already evident at the adenoma stage, characterized by enrichment of *Fusobacterium nucleatum* and reduced abundance of butyrate‐producing bacteria such as *Faecalibacterium prausnitzii* and *Roseburia spp* [63–65]. In the serrated neoplasia pathway, *F. nucleatum* has also been implicated as a key oncobacterium, with a tissue detection rate of approximately 35% in SSLs. Its positivity rate not only increases with the histological grade of SPs but also progressively rises from the sigmoid colon to the cecum in SSLs [66]. Notably, the prevalence of invasive *F. nucleatum* within proximal SSLs (∼79%) is markedly higher than in conventional adenomas (∼29%) [67]. The latest evidence indicates that fecal abundance of *F. nucleatum* may serve as a reliable predictor of metachronous recurrence after endoscopic polypectomy [68]. In addition, gut microbiota dysbiosis can also lead to alterations in metabolic end products, including amino acids. After adenoma removal, the levels of proline, serine, and ornithine in patients decrease to levels similar to those in the control group. Combining microbiota and amino acid expression characteristics may serve as an effective noninvasive strategy for predicting recurrence [69].

### 3.2. Patient Characteristics

#### 3.2.1. General Characteristics

Age at the baseline colonoscopy is an essential factor for the recurrence of colorectal adenoma [45, 70]. A study investigating recurrence following polypectomy revealed that each additional year of age was associated with a 4% increase in the risk of recurrence. Notably, proximal colon lesions exhibited a more pronounced age‐related recurrence trend [71]. The underlying mechanisms remain incompletely understood; however, age‐related immunosenescence, cumulative genomic instability, and prolonged exposure to environmental carcinogens are well‐established contributors to colorectal carcinogenesis in the aging colon [72, 73], which may partly underlie the higher adenoma recurrence rates observed in elderly individuals. In contrast, the prevalence of conventional adenomas increases with age, whereas SSLs show a more stable age‐related trend, suggesting divergent age‐specific biology between the two pathways [74, 75].

Compared to female patients, male patients exhibit earlier and higher recurrence rates in the surveillance following adenoma resection. A large‐scale surveillance cohort study (*n* = 59,667) demonstrated that males had a 10% increased risk of early recurrence, potentially linked to hormonal differences [37]. This finding is further supported by a meta‐analysis of 17 studies (*n* = 924,932) showing that men had significantly elevated risks of advanced neoplasia (pooled RR: 1.83; 95% CI: 1.69–1.97) and CRC (RR: 2.02; 95% CI: 1.53–2.66) compared to women, with consistent associations across all age groups from 40 to over 70 years [76]. Premenopausal women have a higher concentration of estrogen in their bodies, which can play a protective role (such as anti‐inflammation, anti‐oxidation, and regulating the immune microenvironment) [77]. The action of estrogen is mediated through estrogen receptors. Extensive evidence indicates that the expression of estrogen receptor beta is negatively correlated with the presence of adenomas [78]. However, this hormonal advantage may be offset by certain high‐risk features of adenomas at baseline colonoscopy, such as villous architecture and larger adenoma. For this group, the screening interval may be shortened to enhance precise prevention [37]. Notably, the gender predisposition for SPs remains controversial. Unlike conventional adenomas, which predominantly affect males, major studies indicate no significant prevalence difference between genders for SPs [79, 80], while some research supports a female predominance [81]. This divergence may reflect underlying differences in the biological pathways driving each lesion type.

Adenoma recurrence risk exhibits complex race‐ and time‐specific patterns. Conventional wisdom suggests similar overall recurrence rates across racial groups: a prospective study of 1821 patients demonstrated no significant difference in adenoma recurrence between Blacks and Whites (39.2% vs 39.4%; OR: 0.98; 95% CI: 0.80–1.20) [82]. However, a recent large‐scale surveillance cohort study (59,667 adults) has revealed time‐dependent racial disparity patterns: compared with non‐Hispanic Whites, non‐Hispanic Blacks exhibited lower early recurrence risk (aHR: 0.89; 95% CI: 0.83–0.96) and sustained lower mid‐term recurrence risk (5–10 years; aHR: 0.84; 95% CI: 0.77–0.92), with a nonsignificant trend toward higher late recurrence risk (> 10 years; aHR: 1.15; 95% CI: 0.98–1.34); Asian/Pacific Islander patients showed reduced early risk (aHR: 0.80; 95% CI: 0.67–0.96) with subsequent normalization across later time periods [37]. Within the serrated pathway, individuals of White race exhibit a higher risk of SPs compared to African Americans and Hispanics [79, 83]. These findings suggest that racial disparities in adenoma recurrence may reflect complex interactions among genetic susceptibility, environmental exposures, and lifestyle factors, underscoring the importance of developing population‐ and time‐specific individualized surveillance strategies to optimize CRC prevention [84].

#### 3.2.2. Metabolic and Lifestyle Factors

Although current evidence suggests that metabolic syndrome, abdominal obesity, elevated BMI, cigarette smoking, alcohol consumption, and sedentary behavior are shared risk factors for both the serrated and conventional adenoma pathways, the strength of association with each pathway varies for some of these factors [74, 85, 86]. The components of metabolic syndrome, including elevated fasting blood glucose or diabetes, obesity, and dyslipidemia, are closely associated with recurrence [87]. A comprehensive systematic review and meta‐analysis of 17 studies representing 44,336 participants demonstrated that metabolic syndrome patients had a significantly increased risk of colorectal adenoma (OR: 1.39; 95% CI: 1.24–1.57) [88]. Notably, dose–response analysis revealed that every additional metabolic syndrome component was associated with an 8% increment in colorectal adenoma risk (OR: 1.08; 95% CI: 1.04–1.11) [88].

Patients with elevated baseline fasting blood glucose or insulin levels have an increased risk of adenoma recurrence [89]. Higher blood glucose levels increase sensitivity to oxidative stress. Elevated insulin levels affect insulin‐like growth factor (IGF) signaling, leading to increased IGF‐I levels and relatively enhanced biological activity. IGF‐I plays a key role in regulating cell proliferation and apoptosis [90, 91]. This may be a potential mechanism underlying the high recurrence rate of adenomas after resection in patients with impaired glucose tolerance or diabetes. In addition, smoking and drinking habits can also increase the risk of adenoma recurrence by influencing oxidative stress through carcinogens. Carcinogenic substances in tobacco reach the intestinal mucosa through multiple routes, directly disrupting cell replication and impairing DNA repair [92]; smoking is an independent risk factor for adenoma recurrence [93, 94], with risk increasing markedly at exposures of ≥ 35 years or ≥ 30 cigarettes per day [93], and is associated with a higher prevalence of tubular, multiple, larger, and distally located adenomas [95]. Notably, among modifiable risk factors for SPs, smoking shows a particularly strong association―even surpassing that observed for conventional adenomas―with this association being especially pronounced for distal SPs [85, 96]. In contrast, alcohol consumption demonstrates a more consistent and stronger association with the conventional adenoma pathway than with the serrated pathway [96–98]. Alcohol metabolism generates reactive oxygen species via ethanol‐induced upregulation of cytochrome P‐450 2E1 (CYP2E1), and its metabolite acetaldehyde exerts direct carcinogenic and mutagenic effects [99]; large‐scale observational data indicate that drinking alcohol ≥ 2 times per day is associated with the development of colorectal polyps 10 years later [100]. Alcohol consumption also promotes chronic intestinal inflammation by stimulating pro‐inflammatory cytokines and chemokines, as demonstrated in animal models of colon polyp development [101].

Obesity (higher body mass index, BMI) can increase the risk of adenoma recurrence by elevating insulin and IGF‐I levels, and may even promote adenoma progression; this trend is more pronounced in male patients [102, 103]. Sedentary behavior and physical inactivity are primary upstream drivers of this process, as they directly promote visceral fat accumulation and insulin resistance. In male populations, sedentary behavior has been shown to significantly increase the risk of colorectal adenoma recurrence [104]. Conversely, higher levels of physical activity (at least 1 h per week) and lower body fat mass reduce recurrence rates, particularly for AAs [105]. Elevated resting heart rate, a marker of low cardiorespiratory fitness, is also independently associated with AA recurrence, with a stronger association observed in individuals with high body fat or sedentary lifestyles [106]. Long‐term aerobic exercise reduces resting heart rate by increasing cardiac output per beat [107], highlighting the broad metabolic benefits of regular physical activity. Several studies have explored the association between dyslipidemia and the risk of developing colorectal adenomas, but the molecular mechanisms underlying lipid regulation of adenoma recurrence remain unclear. Previous studies have shown that elevated levels of apolipoprotein B and triglycerides are independent risk factors for recurrence following endoscopic resection of adenomas [35]. At least three mechanisms may be involved in this association. One mechanism suggests that lipid abnormalities lead to visceral fat deposition, which is associated with insulin resistance, hyperinsulinemia, and elevated levels of IGF‐I [108]. Another mechanism involves creating a tumorigenic environment by reducing the release of anti‐inflammatory cytokines such as interleukin‐10 and increasing the release of pro‐inflammatory cytokines such as interleukin‐6 and tumor necrosis factor‐α [109]. Finally, existing research suggests that tumor stem cells associated with cancer recurrence may enhance their tumorigenic potential if they are rich in lipid droplets [110, 111].

#### 3.2.3. Dietary Patterns and Nutritional Factors

Dietary pattern analysis offers a more integrative framework for understanding disease risk than individual nutrient assessment alone. The Western dietary pattern, characterized by high intakes of red and processed meat, refined carbohydrates, added sugars, and saturated fat, has been associated with an increased risk of colorectal adenoma in multiple epidemiological studies [112–114]. In contrast, adherence to a Mediterranean dietary pattern—rich in fruits, vegetables, legumes, whole grains, fish, and olive oil—has been associated with a substantially reduced risk of colorectal neoplasia [115]. A dose–response meta‐analysis of 13 prospective cohort studies demonstrated that the highest Mediterranean diet adherence was associated with a significantly lower risk of CRC incidence (summary RR: 0.90; 95% CI: 0.84–0.96), with a 4% risk reduction per 2‐score increment in adherence score [116]. Emerging evidence suggests a similar protective effect at the adenoma stage, though direct evidence from post‐polypectomy cohorts remains limited [117]. In a European intervention trial among patients with prior adenoma resection, Cottet et al. reported that women adhering to a Mediterranean dietary pattern had a significantly reduced risk of 3‐year adenoma recurrence (highest vs. lowest tertile: adjusted OR: 0.30, 95% CI: 0.09–0.98; P for trend = 0.04), whereas no significant association was observed in men; adherence to a Western dietary pattern was not significantly associated with recurrence in either sex [115]. These findings may partly reflect the limited sample size and statistical power of the trial, but it may also suggest that the dietary determinants of adenoma recurrence are more complex than those of initial adenoma development and may exhibit sex‐specific characteristics. Larger studies are needed to confirm these observations.

Among individual dietary components, red and processed meat intake represents the most consistently identified dietary risk factor for adenoma recurrence [118, 119]. Increased red meat intake has been shown to be associated with a higher risk of CRC [120]. In the precancerous stage, it has been reported that the risk of recurrence in patients with multiple or advanced adenomas is positively correlated with the intake of fried, processed, well‐done, or very well‐done red meat. A well‐supported mechanism means specific cooking methods and higher levels of doneness increase the production of heterocyclic amines (which are genotoxic) and other mutagens. These reactive species can trigger covalent modifications of DNA or the formation of DNA adducts at guanine sites, leading to mutations [121]. This association appears even more pronounced within the serrated pathway: the highest quartile of red meat intake was associated with a more than 2.5‐fold increased risk of SSLs (OR: 2.59; 95% CI: 1.41–4.74), significantly exceeding the corresponding risk for conventional adenomas (OR: 1.53; 95% CI: 1.21–1.94; P for difference = 0.003) [122], a finding corroborated by meta‐analytic evidence (OR: 1.30; 95% CI: 1.13–1.51) [123].

Dietary fiber, total fat intake, and added sugars are other key dietary components that can be adjusted. Dietary fiber has been hypothesized to reduce colorectal adenoma risk by diluting fecal carcinogens, shortening colonic transit time, and promoting butyrate production [124, 125]. Within the serrated pathway, fiber intake similarly showed a borderline protective trend that reached significance in meta‐analysis (OR: 0.90; 95% CI: 0.82–0.99) [123]. However, whether this benefit extends to adenoma recurrence remains uncertain. A meta‐analysis of two randomized controlled trials on adenoma prevention (*n* = 1520) found only a weak and statistically insignificant negative correlation between dietary fiber intake and recurrence of all adenomas (RR: 0.85, 95% CI: 0.69–1.05), while this association was stronger for proximal adenomas (RR: 0.73, 95% CI: 0.56–0.97) and specific fiber subtypes (cereals, fruits, and vegetables) [126]. In contrast, a dose–response meta‐analysis of prospective studies found that vegetable and fruit fiber were inversely associated with incident adenoma, but no fiber source was significantly associated with recurrent adenoma [127]. Taken together, stratified analyses in larger prospective cohort studies are still needed. Diets high in total fat and saturated fat are associated with increased bile acid secretion, which promotes colonic epithelial proliferation and may accelerate adenoma development [128, 129]. Elevated baseline serum total bile acid concentrations have been demonstrated to be independently associated with adenoma recurrence risk (OR: 2.17, 95% CI: 1.19–4.04) [130]. In the serrated pathway, although high total fat intake was associated with an approximately threefold increased SSL risk in preliminary analysis (OR: 3.09; 95% CI: 1.24–7.72), this association did not remain statistically significant after further adjustment for red meat intake, suggesting that the observed effect may be largely attributable to red meat consumption [122]. In addition, excessive added sugar intake increases postprandial insulin secretion, thereby indirectly amplifying the previously described insulin/IGF‐1 signaling pathway linked to recurrence [89, 131, 132].

Calcium and vitamin D are among the most extensively studied micronutrients in the chemoprevention of colorectal adenoma recurrence. Calcium inhibits colonic epithelial cell proliferation by binding to secondary bile acids and ionized fatty acids within the intestinal lumen [133]. A systematic review and meta‐analysis of randomized controlled trials demonstrated a statistically significant protective effect of calcium supplementation against adenoma recurrence (RR: 0.89; 95% CI: 0.82–0.96) [134]. Vitamin D, acting via the vitamin D receptor (VDR), regulates colonic cell proliferation, differentiation, and apoptosis, and inverse associations between vitamin D status and colorectal neoplasia risk have been reported in observational studies [135–137]. However, a large randomized trial (*n* = 2259) found no significant reduction in adenoma recurrence with vitamin D3 (1000 IU/day), calcium (1200 mg/day), or both over 3–5 years [138]. Notably, as detailed in the genetic susceptibility section, the GC gene polymorphism (DBP2 isoform, rs4588) substantially modulates the efficacy of both supplements, which may partly account for the inconsistent results observed across trials [139]. Regarding the serrated pathway, the effects of calcium and vitamin D on SSA/Ps remain controversial [140]. Evidence from a large multicenter chemoprevention study even suggested that calcium and vitamin D supplementation (6–10 years after initiation) increased the risk of SSA/Ps [141].

Green tea, rich in catechins, has been investigated as a potential chemopreventive agent against colorectal neoplasia. A prospective cohort study demonstrated that high consumption of green tea (≥ 10 cups/day) was associated with a significant delay in cancer onset, with female and male patients exhibiting delays of 7.3 and 3.2 years, respectively [142]. Subsequently, a randomized clinical trial conducted in a Korean population showed that supplementation with green tea extract (GTE; 0.9 g/day, equivalent to approximately 0.6 g/day of catechins) for 12 months significantly reduced the incidence of metachronous colorectal adenomas compared with controls (23.6% vs. 42.3%; RR: 0.56; 95% CI: 0.34–0.92) [143]. The chemopreventive properties of green tea are primarily attributed to (−)‐epigallocatechin‐3‐gallate (EGCG), its most abundant and bioactive catechin. Mechanistically, EGCG has been shown to induce apoptosis through the modulation of multiple signaling pathways, including the suppression of cyclooxygenase‐2 (COX‐2) expression and the inhibition of insulin‐like growth factor‐1 receptor (IGF‐1R) activation [144, 145]. However, the largest randomized, placebo‐controlled trial to date (the MIRACLE trial; *n* = 879) found no statistically significant reduction in adenoma recurrence with GTE supplementation over 3 years in the overall study population, although a potential benefit was observed in male participants (52.9% vs. 60.4%; *p* = 0.048) [146]. A recent systematic review and meta‐analysis of five RCTs (*n* = 1389) further indicated that long‐term EGCG supplementation was associated with a statistically significant reduction in the recurrence of colorectal neoplasia (*Z* = 2.83; *p* < 0.05) [147]. Collectively, these findings suggest that green tea catechins, particularly EGCG, may hold promise for the chemoprevention of colorectal adenoma recurrence, though further large‐scale trials are warranted to confirm the efficacy, determine the optimal dose and duration, and clarify potential sex‐specific differences in response.

#### 3.2.4. Genetic Susceptibility

Genetic polymorphisms may be an important intrinsic factor influencing colorectal adenoma recurrence. These variants may interact with drugs or dietary exposures to modulate individual susceptibility to recurrence.

In the inflammatory signaling pathway, cytokine and *PTGS2* (COX‐2) gene variants may influence both baseline recurrence risk and responses to chemopreventive agents. In a polyp prevention trial, carriers of the *IL10−1082G > A* variant allele who did not use nonsteroidal anti‐inflammatory drugs (NSAIDs) had a significantly reduced risk of multiple adenoma recurrence. Conversely, among carriers of this genetic polymorphism who used NSAIDs, the risk of recurrence of any type of adenoma increased. This suggests that such patients may not benefit from NSAID chemoprevention [148]. Aspirin, as a nonselective NSAID, reduces the risk of CRC and adenomas by inhibiting COX‐2 activity. A large randomized clinical trial showed that specific *PTGS2* variants independently increase the risk of recurrence. Homozygotes for the minor C allele at the *rs5277* locus had a 51% increased risk of recurrence, while heterozygotes for the minor G allele (*AG* genotype) at the *rs4648310* locus had a 37% increased risk compared to homozygotes for the corresponding common allele. In addition, evidence further suggests that the *rs4648319* polymorphism might influence the efficacy of aspirin therapy [149]. Beyond NSAIDs, the *GC* gene polymorphism at *rs4588* determines vitamin D‐binding protein (DBP) subtype and markedly alters responses to supplementation. Carriers of the DBP2 isoform (*rs4588* ^∗^AC or ^∗^AA) showed reduced adenoma recurrence risk with vitamin D3 and/or calcium, while individuals without DBP2 (*rs4588* ^∗^CC) derived no benefit [139], potentially explaining the inconsistent results of prior supplementation trials [138, 150].

In the Wnt/β‐catenin pathway, a TA haplotype at *APC* codons 486 and 1822 was examined among 1399 participants in two randomized adenoma prevention trials. This haplotype was associated with a 27% reduction in the odds of any metachronous adenoma (OR: 0.73; 95% CI: 0.59–0.91), and carriers of two TA alleles had an 89% lower odds of advanced metachronous adenomas (OR: 0.11; 95% CI: 0.01–0.80) [151]. Downstream of *APC*, the ornithine decarboxylase‐1 (*ODC1*) G316A polymorphism (rs2302615) is a functional modifier of *APC*/*c-Myc*‐dependent tumorigenesis. In adenoma recurrence trials, this variant showed a pronounced gene–aspirin interaction. A‐allele homozygotes using aspirin had approximately one‐tenth the recurrence risk of nonaspirin‐using G‐allele homozygotes, and A‐allele carriers had a 50% reduced risk of AA recurrence with aspirin in the Aspirin/Folate Polyp Prevention Study [152, 153]. The *TCF7L2* rs7903146 T allele was associated with increased colon cancer risk restricted to non‐NSAID users (OR: 1.65; 95% CI: 1.35–2.02), again demonstrating a gene–drug interaction with implications for chemoprevention [154]. Germline variants across multiple Wnt pathway genes have also been shown to predict time to tumor recurrence in colon cancer patients receiving chemotherapy [155], further supporting the role of this pathway in recurrence susceptibility.

Common functional polymorphisms in DNA repair genes may modulate individual susceptibility to colorectal neoplasia by altering the fidelity of DNA replication and damage repair across multiple pathways. In the mismatch repair (MMR) pathway, the *MLH1* promoter variant −93G > A (rs1800734) promotes CpG island hypermethylation, thereby suppressing *MLH1* expression by disrupting the binding of transcription factor AP4 to its promoter consensus sequence [156]. A large case‐control study demonstrated that homozygous carriers of the rs1800734 A allele had a greater than twofold increased risk of microsatellite instability (MSI)‐positive colon cancer compared with GG homozygotes (OR: 2.47; 95% CI: 1.48–4.11). This association was further strengthened by tobacco smoking [157]. Of particular relevance to the serrated pathway, this polymorphism has been shown to exert its pro‐tumorigenic effect preferentially in the context of somatic BRAF V600E mutation—the hallmark early molecular event of serrated carcinogenesis. This implicates rs1800734 as a germline modifier of serrated pathway progression to MSI‐high CRC [158]. Within the same pathway, *MSH6* Gly39Glu (rs1042821) was associated with increased colon cancer risk in men (OR: 1.27; 95% CI: 1.04–1.54) [157]. A subsequent meta‐analysis of 11 studies further demonstrated that the MSH3 Ala1045Thr (rs26279) G > A variant was significantly associated with elevated overall cancer risk. The strongest associations were observed for CRC (allele model, G vs. A: OR: 1.13; 95% CI: 1.05–1.20) [159]. Beyond the MMR pathway, a comprehensive tag‐SNP analysis of 149 DNA repair genes conducted within a large cancer screening trial identified a nonsynonymous variant in the exonuclease gene EXO1 (rs9350, Ser/Phe) as the top‐ranked risk locus for advanced colorectal adenoma (OR: 1.30; 95% CI: 1.11–1.51; *p* = 0.001). Notably, a stronger association was observed among current smokers (OR: 2.15; 95% CI: 1.27–3.65; P‐interaction = 0.002) [160]. It should be noted that the majority of these studies employed incident adenoma or CRC as the primary endpoint rather than metachronous adenoma recurrence following polypectomy. Prospective pharmacogenomic analyses within chemoprevention trials are therefore needed to clarify the clinical relevance of these repair pathway variants in post‐polypectomy surveillance populations.

In the insulin‐IGF signaling pathway, the *IRS1* G972R variant confers a 1.4‐fold increased colon cancer risk, and combined carriership of the *IRS1* R allele and *IGF1* non‐192 allele amplifies risk approximately twofold [161]. A classification tree analysis (a recursive partitioning approach identifying high‐order gene–gene interactions) was applied to 18 IGF/insulin pathway genes pooled from two adenoma chemoprevention trials. The analysis found that the *IGF1R* rs7166348 A allele combined with *PIK3R1* rs1823023 AA genotype conferred the highest probability of metachronous colorectal neoplasia (OR: 3.7; 95% CI: 2.2–6.5), whereas a three‐gene protective combination (*IGF1R* A + *PIK3R1* any G + *INSR* AA) was associated with the lowest probability (OR: 0.22; 95% CI: 0.07–0.66) [162]. These findings suggest that polygenic variation within the insulin‐IGF pathway may contribute to individual differences in adenoma recurrence risk.

### 3.3. Quality of Endoscopic Procedure

#### 3.3.1. Resection Integrity

Compared to surgical procedures, endoscopic treatment offers advantages such as minimal trauma, rapid recovery, and fewer complications, making it the preferred option for treating adenomas. However, there remains a notable risk of recurrence after endoscopic treatment, which varies substantially by lesion type and technique. A multicenter prospective study found that the incomplete resection rate for SSLs was significantly higher than that for conventional adenomas (31.0% vs 7.2%; RR: 3.7) [28]. Endoscopic mucosal resection (EMR), the primary method for polyp removal, can be further categorized into en bloc resection and piecemeal resection. Among these, piecemeal EMR carries a local recurrence risk as high as 20%, significantly higher than the 3% local recurrence risk associated with en bloc EMR [29]. This may be related to residual tissue. To achieve complete resection of large‐diameter, broad‐based lesions, endoscopic submucosal dissection (ESD) was developed. Multiple meta‐analyses have consistently demonstrated the superiority of ESD over EMR in this regard. A pooled analysis of six studies encompassing 1642 lesions showed that ESD achieved significantly higher en bloc resection rates (OR: 7.94; 95% CI: 3.96–15.91) and lower local recurrence rates (OR: 0.09; 95% CI: 0.04–0.19) compared with EMR [163]. A systematic review of 66 studies (17,950 lesions) further confirmed this difference, with recurrence rates of 10.5% (765/7303) for EMR versus 1.3% (50/3910) for ESD (OR: 8.19; 95% CI: 6.2–10.9) [164]. Nevertheless, this superior efficacy comes at the cost of increased adverse events and longer operative times [89]. Recent innovations in the EMR technique have shown promise in narrowing this gap. Meta‐analysis of adjuvant thermal ablation of resection margins, including argon plasma coagulation and snare tip soft coagulation, demonstrated a pooled risk ratio of 0.37 (95% CI: 0.18–0.76) for adenoma recurrence, reducing recurrence from 18.8% to 8.2% [165]. Underwater EMR (UEMR) is an emerging alternative to traditional EMR. It improves the rate of complete resection while maintaining similar safety, thereby reducing the recurrence rate [166]. In summary, resection technique represents a critical modifiable determinant of recurrence, and the choice among EMR, ESD, and their respective modifications should be individualized based on lesion characteristics, operator expertise, and patient factors.

#### 3.3.2. Missed Diagnosis Rate

Most studies on adenoma recurrence do not account for the impact of missed diagnosis rates, resulting in inflated recurrence rates [167]. The missed diagnosis rate for neoplastic polyps during colonoscopy is approximately 12%–26% [167–170]. Back‐to‐back colonoscopy studies demonstrate adenoma miss rates of 17%–24% for all adenomas and 5.4%–6% for advanced adenomas, even under quality‐controlled conditions [168, 171]. A large meta‐analysis of 43 tandem studies (> 15,000 procedures) reported pooled miss rates of 26% (95% CI: 23%–30%) for adenomas and 9% (95% CI: 4%–16%) for advanced adenomas, with higher rates for flat adenomas (34%) and proximal advanced lesions (14%) [170]. Compared to conventional adenomas, SPs are more likely to be missed during colonoscopy due to their subtle endoscopic features, such as flat morphology, indistinct borders, and a mucus cap. In addition, inter‐observer variability in pathological diagnosis—particularly in distinguishing SSLs from HPs—contributes to the challenge. [172–175]. This proves that even patients with normal results during their first colonoscopy need to be followed up. Previous studies have shown that small polyps, multiple polyps, or polyps located in the right half of the colon are more likely to be missed. Adenomas larger than 1 cm are rarely missed [168, 169]. These are closely related to the operator’s technical skills and experience.

Withdrawal time is a critical modifiable quality determinant. The landmark study by Barclay et al. established the 6‐min minimum standard, demonstrating that colonoscopists with withdrawal times ≥ 6 min detected significantly more neoplasia than those with shorter times (28.3% vs. 11.8%, *p* < 0.001) [176]. Recent evidence supports extending this to 9 min. A multicenter RCT of 1027 patients showed that 9 min withdrawal significantly increased adenoma detection versus 6 min withdrawal (36.6% vs. 27.1%, *p* = 0.001), particularly in the proximal colon (21.4% vs. 11.9%, *p* < 0.001) and among less experienced endoscopists (36.8% vs. 23.5%, *p* = 0.001) [177]. These results were further corroborated by a meta‐analysis of seven studies involving 69,551 patients, which confirmed higher adenoma detection with ≥ 9 min versus 6–9 min withdrawal (OR: 1.54; 95% CI: 1.30–1.82) [54]. For patients, inadequate bowel preparation can affect the field of view during the procedure. Therefore, adequate bowel preparation and sufficient withdrawal time are very important for high‐quality colonoscopy.

## 4. Discussion

This review synthesizes updated PPCS guidelines from major gastroenterological societies and examines multifactorial determinants of post‐polypectomy adenoma recurrence.

While the guidelines generally agree that adenoma size, number, and dysplasia grade are key factors in determining surveillance intervals, specific recommendations remain inconsistent. For patients with ≥ 3–4 small tubular adenomas, the USMSTF and Asia‐Pacific consensus recommend repeat colonoscopy at 3–5 years and 3 years, respectively, while ESGE and BSG/ACPGBI/PHE redirect these patients to population‐based screening programs.

There is a discrepancy regarding whether villous histology constitutes a high‐risk feature, irrespective of lesion size. This issue is not specific to small adenomas but applies to conventional adenomas in general. Furthermore, for surveillance after piecemeal resection of adenomas > 20 mm, the USMSTF, ESGE, and BSG all recommend shortening the interval to within 6 months, while the Asia‐Pacific consensus does not provide specific recommendations for this scenario and continues to manage these cases as advanced adenomas (3‐year interval).

Although understanding of serrated pathways has deepened in recent years, surveillance recommendations for SPs remain less clear compared to those for conventional adenomas. Due to insufficient studies specifically evaluating the risk of metachronous advanced neoplasia in SPs, only the USMSTF has provided specific follow‐up recommendations based on lesion number and size. Unfortunately, these are weak recommendations based on low‐quality evidence. To better manage patients with SPs, research in this area should be prioritized.

Regarding risk factors for recurrence, this review analyzes three aspects. First, adenoma characteristics are strong predictors of recurrence, including ≥ 3 adenomas, diameter ≥ 10 mm, HGD, villous histology, and proximal colon location. Second, patient‐specific factors—advanced age, male gender, metabolic disorders, unhealthy lifestyle/dietary habits, supplements, and specific genetic polymorphisms—interact to modulate the risk of recurrence. Third, incomplete resection rates influenced by the resection method and lesion type are significantly associated with increased local recurrence rates. Missed diagnoses may lead to overestimation of recurrence rates. Adequate bowel preparation, sufficient withdrawal time, and endoscopist experience are crucial for reducing the risk of missed diagnoses.

Although SPs share many risk factors with conventional adenomas, the strength of these associations differs between the two lesion types. Among modifiable risk factors, smoking shows a stronger association with SPs than with conventional adenomas. Regarding unmodifiable factors, the role of gender remains controversial. Compared to conventional adenomas, SPs pose greater demands on both endoscopists and pathologists due to their subtle endoscopic features and the challenges in pathological diagnosis, particularly in distinguishing SSLs from HPs.

## 5. Conclusion

In conclusion, this review highlights significant variations among major society PPCS guidelines regarding risk stratification and surveillance strategies, which partly reflect differences in regional healthcare settings. We recommend: (1) improving endoscopic detection and standardizing pathological reporting to reduce missed diagnoses and interobserver variability in the classification of SPs and (2) developing society‐specific guidelines based on local clinical data. Emerging evidence suggests that the gut microbiome and metabolic pathways may play a role in adenoma recurrence, offering promising avenues for noninvasive risk stratification and targeted interventions. Developing evidence‐based risk stratification models that integrate clinical, molecular, and environmental factors is essential for achieving personalized surveillance. This review aims to provide decision‐making support for regions lacking independent guidelines and to help clinicians navigate discrepancies among the existing recommendations.

## Author Contributions

All the authors contributed substantially to discussions of the article content. Jun Yao conceived the idea and revised the manuscript. Xiaojin Tao drafted the manuscript.

## Funding

No funding was received for this research.

## Disclosure

All authors read and approved the final manuscript.

## Conflicts of Interest

The authors declare no conflicts of interest.

## Data Availability

Data sharing is not applicable to this article as no datasets were generated or analyzed during the current study.

## References

[1] Sung H. , Ferlay J. , Siegel R. L. et al., Global Cancer Statistics 2020: GLOBOCAN Estimates of Incidence and Mortality Worldwide for 36 Cancers in 185 Countries, CA: A Cancer Journal for Clinicians. (2021) 71, no. 3, 209–249, 10.3322/caac.21660. 33538338

[2] Suzuki H. , Yamamoto E. , Yamano H.-O. , Nakase H. , and Sugai T. , Integrated Analysis of the Endoscopic, Pathological and Molecular Characteristics of Colorectal Tumorigenesis, Digestion. (2019) 99, no. 1, 33–38, 10.1159/000494410, 2-s2.0-85058811557. 30554192

[3] Jiang C. , Zhou Q. , Yi K. , Yuan Y. , and Xie X. , Colorectal Cancer Initiation: Understanding early-stage Disease for Intervention, Cancer Letters. (2024) 589, 10.1016/j.canlet.2024.216831. 38574882

[4] Zauber A. G. , Winawer S. J. , O’Brien M. J. et al., Colonoscopic Polypectomy and long-term Prevention of colorectal-cancer Deaths, New England Journal of Medicine. (2012) 366, no. 8, 687–696, 10.1056/nejmoa1100370, 2-s2.0-84857392643. 22356322 PMC3322371

[5] Chi Z. , Lin Y. , Huang J. et al., Risk Factors for Recurrence of Colorectal Conventional Adenoma and Serrated Polyp, Gastroenterology Report. (2022) 10, 10.1093/gastro/goab038. PMC8972988 35382162

[6] Gupta S. , Lieberman D. , Anderson J. C. et al., Recommendations for Follow-Up After Colonoscopy and Polypectomy: A Consensus Update by the US Multi-Society Task Force on Colorectal Cancer, American Journal of Gastroenterology. (2020) 115, no. 3, 415–434, 10.14309/ajg.0000000000000544. 32039982 PMC7393611

[7] Hassan C. , Antonelli G. , Dumonceau J.-M. et al., Post-Polypectomy Colonoscopy Surveillance: European Society of Gastrointestinal Endoscopy (ESGE) Guideline-Update 2020, Endoscopy. (2020) 52, no. 08, 687–700, 10.1055/a-1185-3109. 32572858

[8] Md R. , J E. , Cj R. et al., British Society of Gastroenterology/Association of Coloproctology of Great Britain and Ireland/Public Health England Post-polypectomy and Post-colorectal Cancer Resection Surveillance Guidelines, Gut. (2020) 69. 10.1136/gutjnl-2019-319858 PMC6984062 31776230

[9] Sung J. J. Y. , Chiu H.-M. , Lieberman D. et al., Third Asia-Pacific Consensus Recommendations on Colorectal Cancer Screening and Postpolypectomy Surveillance, Gut. (2022) 71, no. 11, 2152–2166, 10.1136/gutjnl-2022-327377. 36002247

[10] Jung Y. S. , Summary and Comparison of Recently Updated Post-Polypectomy Surveillance Guidelines, International Rescuer. (2023) 21, no. 4, 443–451, 10.5217/ir.2023.00107. PMC10626009 37915180

[11] Abu-Freha N. , Katz L. H. , Kariv R. et al., Post-Polypectomy Surveillance Colonoscopy: Comparison of the Updated Guidelines, United European Gastroenterology Journal. (2021) 9, no. 6, 681–687, 10.1002/ueg2.12106. 34077635 PMC8280808

[12] Ibáñez-Sanz G. , Sanz-Pamplona R. , and Garcia M. , Post-Polypectomy Colonoscopy Surveillance: Can we Improve the Diagnostic Yield?, Gastroenterology and Hepatology. (2022) 45, no. 6, 474–487, 10.1016/j.gastrohep.2021.11.005. 34848307

[13] van Heijningen E.-M. B. , Lansdorp-Vogelaar I. , Kuipers E. J. et al., Features of Adenoma and Colonoscopy Associated With Recurrent Colorectal Neoplasia Based on a Large Community-Based Study, Gastroenterology. (2013) 144, no. 7, 1410–1418, 10.1053/j.gastro.2013.03.002, 2-s2.0-84878326308. 23499951

[14] Fairley K. J. , Li J. , Komar M. , Steigerwalt N. , and Erlich P. , Predicting the Risk of Recurrent Adenoma and Incident Colorectal Cancer Based on Findings of the Baseline Colonoscopy, Clinical and Translational Gastroenterology. (2014) 5, no. 12, 10.1038/ctg.2014.11, 2-s2.0-84923358944. PMC4274367 25472702

[15] Jang H. W. , Park S. J. , Hong S. P. , Cheon J. H. , Kim W. H. , and Kim T. I. , Risk Factors for Recurrent High-Risk Polyps After the Removal of High-Risk Polyps at Initial Colonoscopy, Yonsei Medical Journal. (2015) 56, no. 6, 1559–1565, 10.3349/ymj.2015.56.6.1559, 2-s2.0-84944128603. 26446637 PMC4630043

[16] Nagtegaal I. D. , Odze R. D. , Klimstra D. et al., The 2019 WHO Classification of Tumours of the Digestive System, Histopathology. (2020) 76, no. 2, 182–188, 10.1111/his.13975. 31433515 PMC7003895

[17] Crockett S. D. and Nagtegaal I. D. , Terminology, Molecular Features, Epidemiology, and Management of Serrated Colorectal Neoplasia, Gastroenterology. (2019) 157, no. 4, 949–66.e4, 10.1053/j.gastro.2019.06.041, 2-s2.0-85072303618. 31323292

[18] Lieberman D. A. , Rex D. K. , Winawer S. J. , Giardiello F. M. , Johnson D. A. , and Levin T. R. , Guidelines for Colonoscopy Surveillance After Screening and Polypectomy: A Consensus Update by the US Multi-Society Task Force on Colorectal Cancer, Gastroenterology. (2012) 143, no. 3, 844–857, 10.1053/j.gastro.2012.06.001, 2-s2.0-84865514164. 22763141

[19] Hassan C. , Gimeno-García A. , Kalager M. et al., Systematic Review With Meta-Analysis: The Incidence of Advanced Neoplasia After Polypectomy in Patients With and Without low-risk Adenomas, Alimentary Pharmacology & Therapeutics. (2014) 39, no. 9, 905–912, 10.1111/apt.12682, 2-s2.0-84898598352. 24593121

[20] Dubé C. , Yakubu M. , McCurdy B. R. et al., Risk of Advanced Adenoma, Colorectal Cancer, and Colorectal Cancer Mortality in People with Low-Risk Adenomas at Baseline Colonoscopy: A Systematic Review and Meta-Analysis, American Journal of Gastroenterology. (2017) 112, no. 12, 1790–1801, 10.1038/ajg.2017.360, 2-s2.0-85039839975. 29087393

[21] Cairns S. R. , Scholefield J. H. , Steele R. J. et al., Guidelines for Colorectal Cancer Screening and Surveillance in Moderate and High Risk Groups (Update From 2002), Gut. (2010) 59, no. 5, 666–689, 10.1136/gut.2009.179804, 2-s2.0-77951758579. 20427401

[22] Hassan C. , Quintero E. , Dumonceau J.-M. et al., Post-Polypectomy Colonoscopy Surveillance: European Society of Gastrointestinal Endoscopy (ESGE) Guideline, Endoscopy. (2013) 45, no. 10, 842–851, 10.1055/s-0033-1344548, 2-s2.0-84884900478. 24030244

[23] Sung J. J. Y. , Ng S. C. , Chan F. K. L. et al., An Updated Asia Pacific Consensus Recommendations on Colorectal Cancer Screening, Gut. (2015) 64, no. 1, 121–132, 10.1136/gutjnl-2013-306503, 2-s2.0-84919710880. 24647008

[24] Atkin W. , Wooldrage K. , Brenner A. et al., Adenoma Surveillance and Colorectal Cancer Incidence: a Retrospective, Multicentre, Cohort Study, The Lancet Oncology. (2017) 18, no. 6, 823–834, 10.1016/s1470-2045(17)30187-0, 2-s2.0-85018177182. 28457708 PMC5461371

[25] Wieszczy P. , Kaminski M. F. , Franczyk R. et al., Colorectal Cancer Incidence and Mortality After Removal of Adenomas During Screening Colonoscopies, Gastroenterology. (2020) 158, no. 4, 875–83.e5, 10.1053/j.gastro.2019.09.011. 31563625

[26] Foss F. A. , Milkins S. , and McGregor A. H. , Inter-Observer Variability in the Histological Assessment of Colorectal Polyps Detected Through the NHS Bowel Cancer Screening Programme, Histopathology. (2012) 61, no. 1, 47–52, 10.1111/j.1365-2559.2011.04154.x, 2-s2.0-84862868663. 22486166

[27] Mahajan D. , Downs-Kelly E. , Liu X. et al., Reproducibility of the Villous Component and high-grade Dysplasia in Colorectal Adenomas < 1 Cm: Implications for Endoscopic Surveillance, The American Journal of Surgical Pathology. (2013) 37, no. 3, 427–433, 10.1097/pas.0b013e31826cf50f, 2-s2.0-84873992017. 23348206

[28] Pohl H. , Srivastava A. , Bensen S. P. et al., Incomplete Polyp Resection During Colonoscopy-Results of the Complete Adenoma Resection (CARE) Study, Gastroenterology. (2013) 144, no. 1, 74–80.e1, 10.1053/j.gastro.2012.09.043, 2-s2.0-84871323765. 23022496

[29] Belderbos T. D. G. , Leenders M. , Moons L. M. G. , and Siersema P. , Local Recurrence After Endoscopic Mucosal Resection of Nonpedunculated Colorectal Lesions: Systematic Review and meta-analysis, Endoscopy. (2014) 46, no. 05, 388–402, 10.1055/s-0034-1364970, 2-s2.0-84899475404. 24671869

[30] Rex K. D. , Vemulapalli K. C. , and Rex D. K. , Recurrence Rates After EMR of Large Sessile Serrated Polyps, Gastrointestinal Endoscopy. (2015) 82, no. 3, 538–541, 10.1016/j.gie.2015.01.025, 2-s2.0-84939261015. 25851161

[31] Pellise M. , Burgess N. G. , Tutticci N. et al., Endoscopic Mucosal Resection for Large Serrated Lesions in Comparison With Adenomas: A Prospective Multicentre Study of 2000 Lesions, Gut. (2017) 66, no. 4, 644–653, 10.1136/gutjnl-2015-310249, 2-s2.0-84956656827. 26786685

[32] Bertario L. , Russo A. , Sala P. et al., Predictors of Metachronous Colorectal Neoplasms in Sporadic Adenoma Patients, International Journal of Cancer. (2003) 105, no. 1, 82–87, 10.1002/ijc.11036, 2-s2.0-0345701258. 12672034

[33] Viel J.-F. , Studer J.-M. , Ottignon Y. , and Hirsch J. P. , Predictors of Colorectal Polyp Recurrence After the First Polypectomy in Private Practice Settings: a Cohort Study, PLoS One. (2012) 7, no. 12, 10.1371/journal.pone.0050990, 2-s2.0-84870711601. PMC3513321 23226555

[34] Chang J.-J. , Chien C.-H. , Chen S.-W. , Chen L. W. , Liu C. J. , and Yen C. L. , Long Term Outcomes of Colon Polyps With High Grade Dysplasia Following Endoscopic Resection, BMC Gastroenterology. (2020) 20, no. 1, 10.1186/s12876-020-01499-2. PMC7656717 33172387

[35] Du J.-Y. , Huang G.-Y. , Xie Y.-C. , Lin Z. W. , and Zhang L. , High Levels of Triglycerides, Apolipoprotein B, and the Number of Colorectal Polyps are Risk Factors for Colorectal Polyp Recurrence After Endoscopic Resection: A Retrospective Study, Journal of Gastrointestinal Oncology. (2022) 13, no. 4, 1753–1760, 10.21037/jgo-22-491. 36092331 PMC9459209

[36] de Jonge V. , Sint Nicolaas J. , van Leerdam M. E. , Kuipers E. , and Veldhuyzen van Zanten S. , Systematic Literature Review and Pooled Analyses of Risk Factors for Finding Adenomas at Surveillance Colonoscopy, Endoscopy. (2011) 43, no. 07, 560–572, 10.1055/s-0030-1256306, 2-s2.0-79959820289. 21437854

[37] Awan U. A. , Song Q. , Ciombor K. K. et al., Demographic and Clinicopathologic Factors Associated with Colorectal Adenoma Recurrence, JAMA Network Open. (2026) 9, no. 2, 10.1001/jamanetworkopen.2025.56853. PMC12873766 41637071

[38] Anderson J. C. , Morris C. B. , Robertson D. J. et al., Can the Sum of Adenoma Diameters (Adenoma Bulk) on Index Examination Predict Risk of Metachronous Advanced Neoplasia?, Journal of Clinical Gastroenterology. (2018) 52, no. 7, 628–634, 10.1097/mcg.0000000000000899, 2-s2.0-85050307802. 28767463 PMC5794639

[39] Serrano M. , Mão de Ferro S. , Fidalgo P. , Lage P. , Chaves P. , and Dias Pereira A. , Endoscopic Mucosal Resection of Superficial Colorectal Neoplasms: Review of 140 Procedures, Acta Médica Portuguesa. (2012) 25, no. 5, 288–296. 23211198

[40] Baile-Maxía S. , Mangas-Sanjuán C. , Ladabaum U. et al., Risk Factors for Metachronous Colorectal Cancer or Advanced Adenomas After Endoscopic Resection of high-risk Adenomas, Clinical Gastroenterology and Hepatology. (2023) 21, no. 3, 630–643, 10.1016/j.cgh.2022.12.005. 36549471

[41] Djinbachian R. , Lafontaine M. L. , Anderson J. C. et al., Risk of Total Metachronous Advanced Neoplasia at Surveillance Colonoscopy After Detection of Serrated Lesions: a Matched case-cohort Study, Endoscopy. (2023) 55, no. 08, 728–736, 10.1055/a-2020-6797. 36702132

[42] Holme Ø. , Bretthauer M. , Eide T. J. et al., Long-Term Risk of Colorectal Cancer in Individuals With Serrated Polyps, Gut. (2015) 64, no. 6, 929–936, 10.1136/gutjnl-2014-307793, 2-s2.0-84932621321. 25399542

[43] Yoon J. Y. , Kim H. T. , Hong S. P. et al., High-Risk Metachronous Polyps are More Frequent in Patients With Traditional Serrated Adenomas than in Patients With Conventional Adenomas: a Multicenter Prospective Study, Gastrointestinal Endoscopy. (2015) 82, no. 6, 1087–93.e3, 10.1016/j.gie.2015.05.016, 2-s2.0-84952945307. 26117178

[44] Loeve F. , van Ballegooijen M. , Boer R. , Kuipers E. , and Habbema J. , Colorectal Cancer Risk in Adenoma Patients: a Nation-wide Study, International Journal of Cancer. (2004) 111, no. 1, 147–151, 10.1002/ijc.20241, 2-s2.0-3042812643. 15185356

[45] Martínez M. E. , Baron J. A. , Lieberman D. A. et al., A Pooled Analysis of Advanced Colorectal Neoplasia Diagnoses After Colonoscopic Polypectomy, Gastroenterology. (2009) 136, no. 3, 832–841, 10.1053/j.gastro.2008.12.007, 2-s2.0-60449104628. 19171141 PMC3685417

[46] Facciorusso A. , Di Maso M. , Serviddio G. et al., Factors Associated with Recurrence of Advanced Colorectal Adenoma After Endoscopic Resection, Clinical Gastroenterology and Hepatology. (2016) 14, no. 8, 1148–54.e4, 10.1016/j.cgh.2016.03.017, 2-s2.0-84977479666. 27005802

[47] Nusko G. , Hahn E. G. , and Mansmann U. , Characteristics of Metachronous Colorectal Adenomas Found During long-term follow-up: Analysis of Four Subsequent Generations of Adenoma Recurrence, Scandinavian Journal of Gastroenterology. (2009) 44, no. 6, 736–744, 10.1080/00365520902770078, 2-s2.0-67651165548. 19277927

[48] Murakami T. , Mitomi H. , Saito T. et al., Distinct WNT/β-catenin Signaling Activation in the Serrated Neoplasia Pathway and the Adenoma-Carcinoma Sequence of the Colorectum, Modern Pathology. (2015) 28, no. 1, 146–158, 10.1038/modpathol.2014.41, 2-s2.0-84920647010. 24925057

[49] Zhang Y. , Karahalios A. , Aung Y. K. , Win A. K. , Boussioutas A. , and Jenkins M. A. , Risk Factors for Metachronous Colorectal Cancer and Advanced Neoplasia Following Primary Colorectal Cancer: A Systematic Review and Meta-Analysis, BMC Gastroenterology. (2023) 23, no. 1, 10.1186/s12876-023-03053-2. PMC10688466 38036994

[50] Jung Y. S. , Park J. H. , and Park C. H. , Serrated Polyps and the Risk of Metachronous Colorectal Advanced Neoplasia: a Systematic Review and Meta-Analysis, Clinical Gastroenterology and Hepatology. (2022) 20, no. 1, 31–43.e1, 10.1016/j.cgh.2020.09.051. 33007512

[51] Anderson J. C. , Butterly L. F. , Robinson C. M. , Weiss J. E. , Amos C. , and Srivastava A. , Risk of Metachronous High-Risk Adenomas and Large Serrated Polyps in Individuals with Serrated Polyps on Index Colonoscopy: Data from the New Hampshire Colonoscopy Registry, Gastroenterology. (2018) 154, no. 1, 117–27.e2, 10.1053/j.gastro.2017.09.011, 2-s2.0-85040687464. 28927878 PMC5742054

[52] Utsumi T. , Yamada Y. , Diaz-Meco M. T. , Moscat J. , and Nakanishi Y. , Sessile Serrated Lesions With Dysplasia: Is it Possible to Nip Them in the Bud?, Journal of Gastroenterology. (2023) 58, no. 8, 705–717, 10.1007/s00535-023-02003-9. 37219625 PMC10366009

[53] Chen Y. , Polychronidis G. , Zhang Y. et al., Traditional Serrated Adenomas Associated With Risk of Subsequent High-Risk Polyps and Colorectal Cancer, American Journal of Gastroenterology. (2025) . 10.14309/ajg.0000000000003588 40493080

[54] Bhurwal A. , Rattan P. , Sarkar A. et al., A Comparison of 9-min Colonoscopy Withdrawal Time and 6-min Colonoscopy Withdrawal Time: a Systematic Review and meta-analysis, Journal of Gastroenterology and Hepatology. (2021) 36, no. 12, 3260–3267, 10.1111/jgh.15701. 34617312

[55] Martínez M. E. , Sampliner R. , Marshall J. R. , Bhattacharyya A. K. , Reid M. E. , and Alberts D. S. , Adenoma Characteristics as Risk Factors for Recurrence of Advanced Adenomas, Gastroenterology. (2001) 120, no. 5, 1077–1083, 10.1053/gast.2001.23247, 2-s2.0-0035080646. 11266371

[56] Bonithon-Kopp C. , Piard F. , Fenger C. et al., Colorectal Adenoma Characteristics as Predictors of Recurrence, Diseases of the Colon & Rectum. (2004) 47, no. 3, 323–333, 10.1007/s10350-003-0054-1, 2-s2.0-1442275283. 14991494

[57] Laiyemo A. O. , Doubeni C. , Sanderson A. K. et al., Likelihood of Missed and Recurrent Adenomas in the Proximal Versus the Distal Colon, Gastrointestinal Endoscopy. (2011) 74, no. 2, 253–261, 10.1016/j.gie.2011.02.023, 2-s2.0-79960944854. 21549375 PMC3148340

[58] Pohl H. , Robertson D. J. , Mott L. A. et al., Association Between Adenoma Location and Risk of Recurrence, Gastrointestinal Endoscopy. (2016) 84, no. 4, 709–716, 10.1016/j.gie.2016.02.048, 2-s2.0-84963853609. 26975233 PMC5586081

[59] Lee H.-S. , Park H. W. , Lee J.-S. et al., Treatment Outcomes and Recurrence Following Standard Cold Forceps Polypectomy for Diminutive Polyps, Surgical Endoscopy. (2017) 31, no. 1, 159–169, 10.1007/s00464-016-4947-1, 2-s2.0-84976503178. 27369287

[60] Baker F. A. , Gal O. , Tatour M. et al., Serrated Polyps in Colorectal Cancer Prevention: Prevalence, Characteristics and Clinical Insights From a Large Retrospective Cohort Study, BMC Gastroenterology. (2025) 25, no. 1, 10.1186/s12876-025-04129-x. PMC12359896 40826381

[61] Watson K. M. , Gardner I. H. , Anand S. et al., Colonic Microbial Abundances Predict Adenoma Formers, Annals of Surgery. (2023) 277, no. 4, e817–e824, 10.1097/sla.0000000000005261. 35129506 PMC9023594

[62] Yu S. Y. , Xie Y. H. , Qiu Y. W. , Chen Y. , and Fang J. , Moderate Alteration to Gut Microbiota Brought by Colorectal Adenoma Resection, Journal of Gastroenterology and Hepatology. (2019) 34, no. 10, 1758–1765, 10.1111/jgh.14735, 2-s2.0-85067870648. 31115072

[63] Feng Q. , Liang S. , Jia H. et al., Gut Microbiome Development Along the Colorectal adenoma-carcinoma Sequence, Nature Communications. (2015) 6, no. 1, 10.1038/ncomms7528, 2-s2.0-84924690678. 25758642

[64] Yang T. W. , Lee W. H. , Tu S. J. et al., Enterotype-Based Analysis of Gut Microbiota Along the Conventional Adenoma-Carcinoma Colorectal Cancer Pathway, Scientific Reports. (2019) 9, no. 1, 10.1038/s41598-019-45588-z, 2-s2.0-85069903330. PMC6662695 31358825

[65] Wu Y. J. , Xiong J. F. , Zhan C. N. , and Xu H. , Gut Microbiota Alterations in Colorectal adenoma-carcinoma Sequence Based on 16S Rrna Gene Sequencing: a Systematic Review and meta-analysis, Microbial Pathogenesis. (2024) 195, 10.1016/j.micpath.2024.106889. 39197689

[66] Ito M. , Kanno S. , Nosho K. et al., Association of Fusobacterium nucleatum With Clinical and Molecular Features in Colorectal Serrated Pathway, International Journal of Cancer. (2015) 137, no. 6, 1258–1268, 10.1002/ijc.29488, 2-s2.0-84936847682. 25703934

[67] Yu T. , Guo F. , Yu Y. et al., Fusobacterium nucleatum Promotes Chemoresistance to Colorectal Cancer by Modulating Autophagy, Cell. (2017) 170, no. 3, 548–63.e16, 10.1016/j.cell.2017.07.008, 2-s2.0-85026371130. 28753429 PMC5767127

[68] Xue J. H. , Xie Y. H. , Zou T. H. et al., Fecal Fusobacterium nucleatum as a Predictor for Metachronous Colorectal Adenoma After Endoscopic Polypectomy, Journal of Gastroenterology and Hepatology. (2021) 36, no. 10, 2841–2849, 10.1111/jgh.15559. 34121231

[69] Bosch S. , Acharjee A. , Quraishi M. N. et al., The Potential of Fecal Microbiota and Amino Acids to Detect and Monitor Patients With Adenoma, Gut Microbes. (2022) 14, no. 1, 10.1080/19490976.2022.2038863. PMC8865277 35188868

[70] Choi W. S. , Han D. S. , Eun C. S. et al., Three-Year Colonoscopy Surveillance After Polypectomy in Korea: a Korean Association for the Study of Intestinal Diseases (KASID) Multicenter Prospective Study, International Rescuer. (2018) 16, no. 1, 126–133, 10.5217/ir.2018.16.1.126, 2-s2.0-85040678048. PMC5797259 29422807

[71] Pommergaard H.-C. , Burcharth J. , Rosenberg J. , and Raskov H. , Advanced Age is a Risk Factor for Proximal Adenoma Recurrence Following Colonoscopy and Polypectomy, British Journal of Surgery. (2016) 103, no. 2, e100–e105, 10.1002/bjs.10069, 2-s2.0-84955180381. 26667088

[72] Yin Q. , Qin F. , Gan F. et al., Colonic Aging and Colorectal Cancer: An Unignorable Interplay and Its Translational Implications, Biology. (2025) 14, no. 7, 10.3390/biology14070805. PMC12292539 40723364

[73] Zhang T. , Wen R. , Fan H. et al., Impact and Potential Value of Immunosenescence on Solid Gastrointestinal Tumors, Frontiers in Immunology. (2024) 15, 10.3389/fimmu.2024.1375730. PMC11239362 39007138

[74] Lei Y. , Cai Z. , Zheng M. et al., Modifiable Lifestyle and Metabolic Risk Factors for Colorectal Polyps: a Systematic Review and meta-analysis, Frontiers in Public Health. (2025) 13, 10.3389/fpubh.2025.1655750. PMC12542907 41132181

[75] Kim H. Y. , Kim S. M. , Seo J. H. , Park E. H. , Kim N. , and Lee D. H. , Age-Specific Prevalence of Serrated Lesions and Their Subtypes by Screening Colonoscopy: a Retrospective Study, BMC Gastroenterology. (2014) 14, no. 1, 10.1186/1471-230x-14-82, 2-s2.0-84900526219. PMC4021026 24775268

[76] Nguyen S. P. , Bent S. , Chen Y. H. , and Terdiman J. P. , Gender as a Risk Factor for Advanced Neoplasia and Colorectal Cancer: a Systematic Review and meta-analysis, Clinical Gastroenterology and Hepatology. (2009) 7, no. 6, 676–681, 10.1016/j.cgh.2009.01.008, 2-s2.0-67349288090. 19514116

[77] Choi Y. and Kim N. , Sex Difference of Colon Adenoma Pathway and Colorectal Carcinogenesis, Journal of Men’s Health. (2024) 42, no. 2, 256–282, 10.5534/wjmh.230085. PMC10949019 37652658

[78] Williams C. , DiLeo A. , Niv Y. , and Gustafsson J. Å. , Estrogen Receptor Beta as Target for Colorectal Cancer Prevention, Cancer Letters. (2016) 372, no. 1, 48–56, 10.1016/j.canlet.2015.12.009, 2-s2.0-84958213395. 26708506 PMC4744541

[79] Wallace K. , Grau M. V. , Ahnen D. et al., The Association of Lifestyle and Dietary Factors With the Risk for Serrated Polyps of the Colorectum, Cancer Epidemiology, Biomarkers & Prevention. (2009) 18, no. 8, 2310–2317, 10.1158/1055-9965.epi-09-0211, 2-s2.0-68949107404. PMC3669681 19661090

[80] Sanaka M. R. , Gohel T. , Podugu A. et al., Adenoma and Sessile Serrated Polyp Detection Rates: Variation by Patient Sex and Colonic Segment but Not Specialty of the Endoscopist, Diseases of the Colon & Rectum. (2014) 57, 1113–1119, 10.1097/dcr.0000000000000183, 2-s2.0-84907285346. 25101608

[81] Vithayathil M. , Smith S. , and Song M. , Epidemiology of Overall and Early-Onset Serrated Polyps Versus Conventional Adenomas in a Colonoscopy Screening Cohort, International Journal of Cancer. (2023) 152, no. 6, 1085–1094, 10.1002/ijc.34306. 36178673

[82] Laiyemo A. O. , Doubeni C. , Brim H. et al., Short- and Long-Term Risk of Colorectal Adenoma Recurrence Among Whites and Blacks, Gastrointestinal Endoscopy. (2013) 77, no. 3, 447–454, 10.1016/j.gie.2012.11.027, 2-s2.0-84873730785. 23337636 PMC3651852

[83] Wang J. D. , Xu G. S. , Hu X. L. et al., The Histologic Features, Molecular Features, Detection and Management of Serrated Polyps: a Review, Frontiers in Oncology. (2024) 14, 10.3389/fonc.2024.1356250. PMC10955069 38515581

[84] Cruz-Correa M. , The Burden of CRC Among US Hispanics: More Polyps, less Cancer, Digestive Diseases and Sciences. (2017) 62, no. 6, 1396–1398, 10.1007/s10620-017-4531-0, 2-s2.0-85014789873. 28283848

[85] Kim J. , Nath K. , Schmidlin K. et al., Hierarchical Contribution of Individual Lifestyle Factors and Their Interactions on Adenomatous and Serrated Polyp Risk, Journal of Gastroenterology. (2023) 58, no. 9, 856–867, 10.1007/s00535-023-02004-8. 37300599 PMC10423128

[86] Ye X. , Han P. , Wu Z. et al., New Management of Surveillance in Patients With Baseline Serrated Polyps: a Large single-center Retrospective Cohort Study in China, European Journal of Gastroenterology and Hepatology. (2023) 35, no. 2, 181–190, 10.1097/meg.0000000000002494. 36574309

[87] Punthakee Z. , Goldenberg R. , and Katz P. , Definition, Classification and Diagnosis of Diabetes, Prediabetes and Metabolic Syndrome, Canadian Journal of Diabetes. (2018) 42, S10–S15, 10.1016/j.jcjd.2017.10.003, 2-s2.0-85044748604. 29650080

[88] Wu H. , Zhang J. , and Zhou B. , Metabolic Syndrome and Colorectal Adenoma Risk: a Systematic Review and meta-analysis, Clinics and Research in Hepatology and Gastroenterology. (2021) 45, no. 5, 10.1016/j.clinre.2021.101749. 34214702

[89] Flood A. , Mai V. , Pfeiffer R. et al., Elevated Serum Concentrations of Insulin and Glucose Increase Risk of Recurrent Colorectal Adenomas, Gastroenterology. (2007) 133, no. 5, 1423–1429, 10.1053/j.gastro.2007.08.040, 2-s2.0-35649013300. 17904132

[90] Sandhu M. S. , Insulin, Insulin-like Growth Factor-I (IGF-I), IGF Binding Proteins, Their Biologic Interactions, and Colorectal Cancer, Journal of the National Cancer Institute. (2002) 94, no. 13, 972–980, 10.1093/jnci/94.13.972. 12096082

[91] Pollak M. N. , Schernhammer E. S. , and Hankinson S. E. , Insulin-Like Growth Factors and Neoplasia, Nature Reviews Cancer. (2004) 4, no. 7, 505–518, 10.1038/nrc1387, 2-s2.0-3042781558. 15229476

[92] Yamasaki E. and Ames B. N. , Concentration of Mutagens From Urine by Adsorption With the Nonpolar Resin XAD-2: Cigarette Smokers Have Mutagenic Urine, Proceedings of the National Academy of Sciences of the United States of America. (1977) 74, no. 8, 3555–3559, 10.1073/pnas.74.8.3555, 2-s2.0-0000545789. 333441 PMC431630

[93] Reid M. E. , Marshall J. R. , Roe D. et al., Smoking Exposure as a Risk Factor for Prevalent and Recurrent Colorectal Adenomas, Cancer Epidemiology, Biomarkers & Prevention. (2003) 12, no. 10, 1006–1011. 14578135

[94] Lee W. C. , Neugut A. I. , Garbowski G. C. et al., Cigarettes, Alcohol, Coffee, and Caffeine as Risk Factors for Colorectal Adenomatous Polyps, Annals of Epidemiology. (1993) 3, 239–244, 10.1016/1047-2797(93)90025-y, 2-s2.0-0027173562. 8275195

[95] Hu N.-C. , Chen J.-D. , Lin Y.-M. , Chang J. Y. , and Chen Y. H. , Stepwise Relationship Between Components of Metabolic Syndrome and Risk of Colorectal Adenoma in a Taiwanese Population Receiving Screening Colonoscopy, Journal of the Formosan Medical Association. (2011) 110, no. 2, 100–108, 10.1016/s0929-6646(11)60016-8, 2-s2.0-79955596602. 21377064

[96] Bailie L. , Loughrey M. B. , and Coleman H. G. , Lifestyle Risk Factors for Serrated Colorectal Polyps: A Systematic Review and meta-analysis, Gastroenterology. (2017) 152, no. 1, 92–104, 10.1053/j.gastro.2016.09.003, 2-s2.0-85006321116. 27639804

[97] Xu J. , Chi P. , Qin K. et al., Association Between Lifestyle and Dietary Preference Factors and Conventional Adenomas and Serrated Polyps, Frontiers in Nutrition. (2023) 10, 10.3389/fnut.2023.1269629. PMC10806101 38268677

[98] Wang Y. M. , Zhou Q. Y. , Zhu J. Z. , Zhu K. F. , Yu C. H. , and Li Y. M. , Systematic Review With Meta-Analysis: Alcohol Consumption and Risk of Colorectal Serrated Polyp, Digestive Diseases and Sciences. (2015) 60, no. 7, 1889–1902, 10.1007/s10620-014-3518-3, 2-s2.0-84930823595. 25618311

[99] Rumgay H. , Murphy N. , Ferrari P. , and Soerjomataram I. , Alcohol and Cancer: Epidemiology and Biological Mechanisms, Nutrients. (2021) 13, no. 9, 10.3390/nu13093173. PMC8470184 34579050

[100] Kahn H. S. , Tatham L. M. , Thun M. J. , and Heath C. W. , Risk Factors for Self-Reported Colon Polyps, Journal of General Internal Medicine. (1998) 13, no. 5, 303–310, 10.1046/j.1525-1497.1998.00095.x, 2-s2.0-0031780726. 9613885 PMC1496952

[101] Shukla P. K. , Chaudhry K. K. , Mir H. et al., Chronic Ethanol Feeding Promotes Azoxymethane and Dextran Sulfate sodium-induced Colonic Tumorigenesis Potentially by Enhancing Mucosal Inflammation, BMC Cancer. (2016) 16, no. 1, 10.1186/s12885-016-2180-x, 2-s2.0-84980417636. PMC4782373 26951793

[102] Jacobs E. T. , Ahnen D. J. , Ashbeck E. L. et al., Association Between Body Mass Index and Colorectal Neoplasia at follow-up Colonoscopy: a Pooling Study, American Journal of Epidemiology. (2009) 169, no. 6, 657–666, 10.1093/aje/kwn401, 2-s2.0-61449136888. 19147743 PMC2727215

[103] Jung Y. S. , Park J. H. , Park D. I. , Sohn C. I. , and Choi K. , Weight Change and Obesity Are Associated With a Risk of Adenoma Recurrence, Digestive Diseases and Sciences. (2016) 61, no. 9, 2694–2703, 10.1007/s10620-016-4194-2, 2-s2.0-84970028687. 27193563

[104] Sardo Molmenti C. L. , Hibler E. A. , Ashbeck E. L. et al., Sedentary Behavior is Associated With Colorectal Adenoma Recurrence in Men, Cancer Causes & Control. (2014) 25, no. 10, 1387–1395, 10.1007/s10552-014-0444-9, 2-s2.0-84911006071. 25060482 PMC4228938

[105] Park J. , Kim J. H. , Lee H. J. et al., The Effects of Physical Activity and Body Fat Mass on Colorectal Polyp Recurrence in Patients With Previous Colorectal Cancer, Cancer Prevention Research. (2017) 10, no. 8, 478–484, 10.1158/1940-6207.capr-17-0065, 2-s2.0-85027035097. 28584169

[106] Park J. , Kim J. H. , Park Y. et al., Resting Heart Rate is an Independent Predictor of Advanced Colorectal Adenoma Recurrence, PLoS One. (2018) 13, no. 3, 10.1371/journal.pone.0193753, 2-s2.0-85042873170. PMC5834177 29499053

[107] Oh D.-J. , Hong H.-O. , and Lee B.-A. , The Effects of Strenuous Exercises on Resting Heart Rate, Blood Pressure, and Maximal Oxygen Uptake, Journal of Exercise Rehabilitation. (2016) 12, no. 1, 42–46, 10.12965/jer.150258. 26933659 PMC4771152

[108] Taniguchi L. , Higurashi T. , Uchiyama T. et al., Metabolic Factors Accelerate Colorectal Adenoma Recurrence, BMC Gastroenterology. (2014) 14, no. 1, 10.1186/1471-230x-14-187, 2-s2.0-84964312674. PMC4287308 25341954

[109] Esteve E. , Ricart W. , and Fernández-Real J. M. , Dyslipidemia and Inflammation: An Evolutionary Conserved Mechanism, Clinical Nutrition. (2005) 24, no. 1, 16–31, 10.1016/j.clnu.2004.08.004, 2-s2.0-12744263365. 15681098

[110] Mutoh M. , Komiya M. , Teraoka N. et al., Overexpression of Low‐Density Lipoprotein Receptor and Lipid Accumulation in Intestinal Polyps in Min Mice, International Journal of Cancer. (2009) 125, no. 11, 2505–2510, 10.1002/ijc.24667, 2-s2.0-70350736113. 19544529

[111] Tirinato L. , Liberale C. , Di Franco S. et al., Lipid Droplets: a New Player in Colorectal Cancer Stem Cells Unveiled by Spectroscopic Imaging, Stem Cells. (2015) 33, no. 1, 35–44, 10.1002/stem.1837, 2-s2.0-84919429576. 25186497 PMC4311668

[112] Fung T. T. , Rimm E. B. , Spiegelman D. et al., Association Between Dietary Patterns and Plasma Biomarkers of Obesity and Cardiovascular Disease Risk, American Journal of Clinical Nutrition. (2001) 73, no. 1, 61–67, 10.1093/ajcn/73.1.61. 11124751

[113] Randi G. , Edefonti V. , Ferraroni M. , La Vecchia C. , and Decarli A. , Dietary Patterns and the Risk of Colorectal Cancer and Adenomas, Nutrition Reviews. (2010) 68, no. 7, 389–408, 10.1111/j.1753-4887.2010.00299.x, 2-s2.0-77956628672. 20591107

[114] Godos J. , Bella F. , Torrisi A. , Sciacca S. , Galvano F. , and Grosso G. , Dietary Patterns and Risk of Colorectal Adenoma: a Systematic Review and Meta-Analysis of Observational Studies, Journal of Human Nutrition and Dietetics. (2016) 29, no. 6, 757–767, 10.1111/jhn.12395, 2-s2.0-84978284939. 27412573

[115] Cottet V. , Bonithon-Kopp C. , Kronborg O. et al., Dietary Patterns and the Risk of Colorectal Adenoma Recurrence in a European Intervention Trial, European Journal of Cancer Prevention. (2005) 14, no. 1, 21–29, 10.1097/00008469-200502000-00004, 2-s2.0-15444370305. 15677892

[116] Zhong Y. , Zhu Y. , Li Q. et al., Association Between Mediterranean Diet Adherence and Colorectal Cancer: a dose-response meta-analysis, American Journal of Clinical Nutrition. (2020) 111, no. 6, 1214–1225, 10.1093/ajcn/nqaa083. 32359135

[117] Jafari N. S. , Clark C. C. T. , and Entezari M. , Mediterranean Diet and Colorectal Adenomas: a Systematic Review and meta-analysis of Observational Studies, European Journal of Cancer Prevention. (2024) 33, no. 3, 223–231, 10.1097/CEJ.0000000000000861. 37942952

[118] Aune D. , Chan D. S. , Vieira A. R. et al., Red and Processed Meat Intake and Risk of Colorectal Adenomas: a Systematic Review and meta-analysis of Epidemiological Studies, Cancer Causes & Control. (2013) 24, no. 4, 611–627, 10.1007/s10552-012-0139-z, 2-s2.0-84876496681. 23380943

[119] Zhao Z. , Yin Z. , Hang Z. , Zhang C. , and Zhao Q. , Association Between Red and Processed Meat Intake and Colorectal Adenoma Incidence and Recurrence: A Systematic Review and Meta-Analysis, Oncotarget. (2018) 9, no. 64, 32373–32382, 10.18632/oncotarget.23561, 2-s2.0-85051762352. 30190793 PMC6122348

[120] Larsson S. C. and Wolk A. , Meat Consumption and Risk of Colorectal Cancer: a Meta‐Analysis of Prospective Studies, International Journal of Cancer. (2006) 119, no. 11, 2657–2664, 10.1002/ijc.22170, 2-s2.0-33750617498. 16991129

[121] Martinez M. E. , Jacobs E. T. , Ashbeck E. L. et al., Meat Intake, Preparation Methods, Mutagens and Colorectal Adenoma Recurrence, Carcinogenesis. (2007) 28, no. 9, 2019–2027, 10.1093/carcin/bgm179, 2-s2.0-34848914086. 17690112

[122] Davenport J. R. , Su T. , Zhao Z. et al., Modifiable Lifestyle Factors Associated With Risk of Sessile Serrated Polyps, Conventional Adenomas and Hyperplastic Polyps, Gut. (2018) 67, no. 3, 456–465, 10.1136/gutjnl-2016-312893, 2-s2.0-85001858044. 27852795 PMC5432410

[123] Zhu Z. , Guan X. , Liu N. , Zhu X. , Dai S. , and Xiong D. , Association Between Dietary Factors and Colorectal Serrated Polyps: a Systematic Review and meta-analysis, Frontiers in Nutrition. (2023) 10, 10.3389/fnut.2023.1187539. PMC10413578 37575321

[124] Coleman H. G. , Murray L. J. , Hicks B. et al., Dietary Fiber and the Risk of Precancerous Lesions and Cancer of the Esophagus: a Systematic Review and meta-analysis, Nutrition Reviews. (2013) 71, no. 7, 474–482, 10.1111/nure.12032, 2-s2.0-84879776725. 23815145

[125] Nucci D. , Fatigoni C. , Salvatori T. , Nardi M. , Realdon S. , and Gianfredi V. , Association Between Dietary Fibre Intake and Colorectal Adenoma: a Systematic Review and Meta-Analysis, International Journal of Environmental Research and Public Health. (2021) 18, no. 8, 10.3390/ijerph18084168. PMC8071151 33920845

[126] Robertson D. J. , Sandler R. S. , Haile R. et al., Fat, Fiber, Meat and the Risk of Colorectal Adenomas, American Journal of Gastroenterology. (2005) 100, no. 12, 2789–2795, 10.1111/j.1572-0241.2005.00336.x, 2-s2.0-33644823438. 16393237

[127] Oh H. , Kim H. , Lee D. H. et al., Different Dietary Fibre Sources and Risks of Colorectal Cancer and Adenoma: A Dose-Response Meta-Analysis of Prospective Studies, The British Journal of Nutrition. (2019) 122, no. 6, 605–615, 10.1017/s0007114519001454, 2-s2.0-85068346504. 31495339

[128] Stadler J. , Stern H. S. , Yeung K. S. et al., Effect of High Fat Consumption on Cell Proliferation Activity of Colorectal Mucosa and on Soluble Faecal Bile Acids, Gut. (1988) 29, no. 10, 1326–1331, 10.1136/gut.29.10.1326, 2-s2.0-0023795233. 3197978 PMC1434012

[129] Liu Y. , Zhang S. , Zhou W. , Hu D. , Xu H. , and Ji G. , Secondary Bile Acids and Tumorigenesis in Colorectal Cancer, Frontiers in Oncology. (2022) 12, 10.3389/fonc.2022.813745. PMC9097900 35574393

[130] Byrd D. A. , Gomez M. , Hogue S. et al., Circulating Bile Acids and Adenoma Recurrence in the Context of Adherence to a High-Fiber, High-Fruit and Vegetable, and Low-Fat Dietary Intervention, Clinical and Translational Gastroenterology. (2022) 13, no. 10, 10.14309/ctg.0000000000000533. PMC9624497 36113023

[131] Giovannucci E. , Insulin, Insulin-like Growth Factors and Colon Cancer: A Review of the Evidence, The Journal of Nutrition. (2001) 131, no. 11, 3109s–3120s, 10.1093/jn/131.11.3109s. 11694656

[132] Pollak M. , Insulin and Insulin-Like Growth Factor Signalling in Neoplasia, Nature Reviews Cancer. (2008) 8, no. 12, 915–928, 10.1038/nrc2536, 2-s2.0-56749184290. 19029956

[133] Baron J. A. , Beach M. , Mandel J. S. et al., Calcium Supplements for the Prevention of Colorectal Adenomas. Calcium Polyp Prevention Study Group, New England Journal of Medicine. (1999) 340, no. 2, 101–107, 10.1056/nejm199901143400204, 2-s2.0-0033552894. 9887161

[134] Bonovas S. , Fiorino G. , Lytras T. , Malesci A. , and Danese S. , Calcium Supplementation for the Prevention of Colorectal Adenomas: a Systematic Review and Meta-Analysis of Randomized Controlled Trials, World Journal of Gastroenterology. (2016) 22, no. 18, 4594–4603, 10.3748/wjg.v22.i18.4594, 2-s2.0-84971010977. 27182169 PMC4858641

[135] Grau M. V. , Baron J. A. , Sandler R. S. et al., Vitamin D, Calcium Supplementation, and Colorectal Adenomas: Results of a Randomized Trial, Journal of the National Cancer Institute. (2003) 95, no. 23, 1765–1771, 10.1093/jnci/djg110. 14652238

[136] Wei M. Y. , Garland C. F. , Gorham E. D. , Mohr S. B. , and Giovannucci E. , Vitamin D and Prevention of Colorectal Adenoma: A Meta-Analysis, Cancer Epidemiology, Biomarkers & Prevention. (2008) 17, no. 11, 2958–2969, 10.1158/1055-9965.epi-08-0402, 2-s2.0-55849124377. 18990737

[137] Ma Y. , Zhang P. , Wang F. , Yang J. , Liu Z. , and Qin H. , Association Between Vitamin D and Risk of Colorectal Cancer: a Systematic Review of Prospective Studies, Journal of Clinical Oncology. (2011) 29, no. 28, 3775–3782, 10.1200/jco.2011.35.7566, 2-s2.0-80053636651. 21876081

[138] Baron J. A. , Barry E. L. , Mott L. A. et al., A Trial of Calcium and Vitamin D for the Prevention of Colorectal Adenomas, New England Journal of Medicine. (2015) 373, no. 16, 1519–1530, 10.1056/nejmoa1500409, 2-s2.0-84944463603. 26465985 PMC4643064

[139] Gibbs D. C. , Barry E. L. , Fedirko V. , Baron J. A. , and Bostick R. M. , Impact of Common Vitamin D-Binding Protein Isoforms on Supplemental Vitamin D3 and/or Calcium Effects on Colorectal Adenoma Recurrence Risk: a Secondary Analysis of a Randomized Clinical Trial, JAMA Oncology. (2023) 9, no. 4, 546–551, 10.1001/jamaoncol.2022.6924. 36701139 PMC9880863

[140] García-Morales N. , Satorres C. , and Bustamante-Balén M. , Calcium and Vitamin D in the Serrated Neoplastic Pathway: Friends or Foes?, World Journal of Gastrointestinal Pathophysiology. (2018) 9, no. 3, 59–62, 10.4291/wjgp.v9.i3.59. 30386666 PMC6209580

[141] Crockett S. D. , Barry E. L. , Mott L. A. et al., Calcium and Vitamin D Supplementation and Increased Risk of Serrated Polyps: Results From a Randomised Clinical Trial, Gut. (2019) 68, no. 3, 475–486, 10.1136/gutjnl-2017-315242, 2-s2.0-85049140913. 29496722 PMC6286251

[142] Shimizu M. , Fukutomi Y. , Ninomiya M. et al., Green Tea Extracts for the Prevention of Metachronous Colorectal Adenomas: a Pilot Study, Cancer Epidemiology, Biomarkers & Prevention. (2008) 17, no. 11, 3020–3025, 10.1158/1055-9965.epi-08-0528, 2-s2.0-55849121192. 18990744

[143] Shin C. M. , Lee D. H. , Seo A. Y. et al., Green Tea Extracts for the Prevention of Metachronous Colorectal Polyps Among Patients Who Underwent Endoscopic Removal of Colorectal Adenomas: a Randomized Clinical Trial, Clinical Nutrition. (2018) 37, no. 2, 452–458, 10.1016/j.clnu.2017.01.014, 2-s2.0-85012250808. 28209333

[144] Shimizu M. , Deguchi A. , Hara Y. , Moriwaki H. , and Weinstein I. B. , EGCG Inhibits Activation of the Insulin-like Growth factor-1 Receptor in Human Colon Cancer Cells, Biochemical and Biophysical Research Communications. (2005) 334, no. 3, 947–953, 10.1016/j.bbrc.2005.06.182, 2-s2.0-22744444072. 16053920

[145] Park I. J. , Lee Y. K. , Hwang J. T. , Kwon D. , Ha J. , and Park O. J. , Green Tea Catechin Controls Apoptosis in Colon Cancer Cells by Attenuation of H_2_O_2_‐Stimulated COX‐2 Expression via the AMPK Signaling Pathway at Low‐Dose H_2_O_2_ , Annals of the New York Academy of Sciences. (2009) 1171, no. 1, 538–544, 10.1111/j.1749-6632.2009.04698.x, 2-s2.0-69149091648. 19723101

[146] Seufferlein T. , Ettrich T. J. , Menzler S. et al., Green Tea Extract to Prevent Colorectal Adenomas, Results of a Randomized, Placebo-Controlled Clinical Trial, American Journal of Gastroenterology. (2022) 117, no. 6, 884–894, 10.14309/ajg.0000000000001706. 35213393

[147] He B. , Kang S. , Su R. et al., Chemoprophylaxis Effect of EGCG on the Recurrence of Colorectal Cancer: a Systematic Review and Meta-Analysis, Current Pharmaceutical Design. (2024) 30, no. 33, 2643–2651, 10.2174/0113816128319678240612114820. 38988171

[148] Sansbury L. B. , Bergen A. W. , Wanke K. L. et al., Inflammatory Cytokine Gene Polymorphisms, Nonsteroidal Anti-Inflammatory Drug Use, and Risk of Adenoma Polyp Recurrence in the Polyp Prevention Trial, Cancer Epidemiology, Biomarkers & Prevention. (2006) 15, no. 3, 494–501, 10.1158/1055-9965.epi-05-0763, 2-s2.0-33645500758. 16537707

[149] Barry E. L. , Sansbury L. B. , Grau M. V. et al., Cyclooxygenase-2 Polymorphisms, Aspirin Treatment, and Risk for Colorectal Adenoma Recurrence--Data From a Randomized Clinical Trial, Cancer Epidemiology, Biomarkers & Prevention. (2009) 18, no. 10, 2726–2733, 10.1158/1055-9965.epi-09-0363, 2-s2.0-70350101185. PMC2769932 19755647

[150] Lazzeroni M. , Serrano D. , Pilz S. et al., Vitamin D Supplementation and Cancer: Review of Randomized Controlled Trials. 23094929

[151] Egan J. B. , Jacobs E. T. , Martínez M. E. , Gerner E. W. , Jurutka P. W. , and Thompson P. A. , Presence of a TA Haplotype in the APC Gene Containing the Common 1822 Polymorphism and Colorectal Adenoma, Cancer Research. (2008) 68, no. 14, 6006–6013, 10.1158/0008-5472.can-08-1084, 2-s2.0-48649092468. 18632657

[152] Martinez M. E. , O’Brien T. G. , Fultz K. E. et al., Pronounced Reduction in Adenoma Recurrence Associated With Aspirin Use and a Polymorphism in the Ornithine Decarboxylase Gene, Proceedings of the National Academy of Sciences of the United States of America. (2003) 100, no. 13, 7859–7864, 10.1073/pnas.1332465100, 2-s2.0-0037934362. 12810952 PMC164678

[153] Barry E. L. , Baron J. A. , Bhat S. et al., Ornithine Decarboxylase Polymorphism Modification of Response to Aspirin Treatment for Colorectal Adenoma Prevention, Journal of the National Cancer Institute. (2006) 98, no. 20, 1494–1500, 10.1093/jnci/djj398, 2-s2.0-33750205691. 17047198

[154] Slattery M. L. , Folsom A. R. , Wolff R. , Herrick J. , Caan B. J. , and Potter J. D. , Transcription Factor 7-like 2 Polymorphism and Colon Cancer, Cancer Epidemiology, Biomarkers & Prevention. (2008) 17, no. 4, 978–982, 10.1158/1055-9965.epi-07-2687, 2-s2.0-42149181801. PMC2587179 18398040

[155] Páez D. , Gerger A. , Zhang W. et al., Association of Common Gene Variants in the WNT/β-catenin Pathway With Colon Cancer Recurrence, The Pharmacogenomics Journal. (2014) 14, no. 2, 142–150, 10.1038/tpj.2013.20, 2-s2.0-84897409111. 23817222

[156] Thomas R. , Trapani D. , Goodyer-Sait L. et al., The Polymorphic Variant rs1800734 Influences Methylation Acquisition and Allele-specific TFAP4 Binding in the MLH1 Promoter Leading to Differential mRNA Expression, Scientific Reports. (2019) 9, no. 1, 10.1038/s41598-019-49952-x, 2-s2.0-85072282286. PMC6748923 31530880

[157] Campbell P. T. , Curtin K. , Ulrich C. M. et al., Mismatch Repair Polymorphisms and Risk of Colon Cancer, Tumour Microsatellite Instability and Interactions With Lifestyle Factors, Gut. (2009) 58, no. 5, 661–667, 10.1136/gut.2007.144220, 2-s2.0-66349113849. 18523027 PMC2903215

[158] De Palma F. D. E. , D’Argenio V. , Pol J. et al., The Molecular Hallmarks of the Serrated Pathway in Colorectal Cancer, Cancers (Basel). (2019) 11. 10.3390/cancers11071017 PMC6678087 31330830

[159] Miao H. K. , Chen L. P. , Cai D. P. , Kong W. J. , and Xiao L. , MSH3 rs26279 Polymorphism Increases Cancer Risk: a meta-analysis, International Journal of Clinical and Experimental Pathology. (2015) 8, no. 9, 11060–11067. 26617824 PMC4637639

[160] Gao Y. , Hayes R. B. , Huang W. Y. et al., DNA Repair Gene Polymorphisms and Tobacco Smoking in the Risk for Colorectal Adenomas, Carcinogenesis. (2011) 32, no. 6, 882–887, 10.1093/carcin/bgr071, 2-s2.0-79958199282. 21504893 PMC3106441

[161] Slattery M. L. , Samowitz W. , Curtin K. et al., Associations Among IRS1, IRS2, IGF1, and IGFBP3 Genetic Polymorphisms and Colorectal Cancer, Cancer Epidemiology, Biomarkers & Prevention. (2004) 13, no. 7, 1206–1214, 10.1158/1055-9965.1206.13.7. 15247132

[162] LeRoy E. C. , Moore J. H. , Hu C. et al., Genes in the Insulin and Insulin-like Growth Factor Pathway and Odds of Metachronous Colorectal Neoplasia, Human Genetics. (2011) 129, no. 5, 503–512, 10.1007/s00439-010-0942-0, 2-s2.0-79955566669. 21221997

[163] Wang J. , Zhang X. H. , Ge J. , Yang C. M. , Liu J. Y. , and Zhao S. L. , Endoscopic Submucosal Dissection Vs Endoscopic Mucosal Resection for Colorectal Tumors: A Meta-Analysis, World Journal of Gastroenterology. (2014) 20, no. 25, 8282–8287, 10.3748/wjg.v20.i25.8282, 2-s2.0-84903934285. 25009404 PMC4081704

[164] De Ceglie A. , Hassan C. , Mangiavillano B. et al., Endoscopic Mucosal Resection and Endoscopic Submucosal Dissection for Colorectal Lesions: a Systematic Review, Critical Reviews in Oncology. (2016) 104, 138–155, 10.1016/j.critrevonc.2016.06.008, 2-s2.0-84979697066. 27370173

[165] Kemper G. , Turan A. S. , Schoon E. J. et al., Endoscopic Techniques to Reduce Recurrence Rates After Colorectal EMR: Systematic Review and Meta-Analysis, Surgical Endoscopy. (2021) 35, no. 10, 5422–5429, 10.1007/s00464-021-08574-z. 34076765 PMC8437853

[166] de Souza M. H. G. , do Espirito Santo P. A. , Maluf-Filho F. , and Lenz L. , Underwater Versus Conventional Endoscopic Mucosal Resection for Colorectal Lesions: a Systematic Review and Meta-Analysis of Randomized Clinical Trials, International Journal of Colorectal Disease. (2023) 38, no. 1, 10.1007/s00384-023-04505-7. 37552342

[167] Ji J.-S. , Choi K.-Y. , Lee W.-C. et al., Endoscopic and Histopathologic Predictors of Recurrence of Colorectal Adenoma on Lowering the Miss Rate, Korean Journal of Internal Medicine (English Edition). (2009) 24, no. 3, 196–202, 10.3904/kjim.2009.24.3.196, 2-s2.0-70449725263. PMC2732778 19721855

[168] Rex D. , Cutler C. , Lemmel G. et al., Colonoscopic Miss Rates of Adenomas Determined by Back-to-Back Colonoscopies, Gastroenterology. (1997) 112, no. 1, 24–28, 10.1016/s0016-5085(97)70214-2, 2-s2.0-0031022236. 8978338

[169] Bensen S. , Mott L. A. , Dain B. , Rothstein R. , and Baron J. , The Colonoscopic Miss Rate and True One-Year Recurrence of Colorectal Neoplastic Polyps, American Journal of Gastroenterology. (1999) 94, no. 1, 194–199, 10.1111/j.1572-0241.1999.00796.x, 2-s2.0-0032894929. 9934755

[170] Zhao S. , Wang S. , Pan P. et al., Magnitude, Risk Factors, and Factors Associated with Adenoma Miss Rate of Tandem Colonoscopy: a Systematic Review and meta-analysis, Gastroenterology. (2019) 156, no. 6, 1661–74.e11, 10.1053/j.gastro.2019.01.260, 2-s2.0-85064319977. 30738046

[171] Ahn S. B. , Han D. S. , Bae J. H. , Byun T. J. , Kim J. P. , and Eun C. S. , The Miss Rate for Colorectal Adenoma Determined by Quality-Adjusted, Back-to-Back Colonoscopies, Gut and Liver. (2012) 6, no. 1, 64–70, 10.5009/gnl.2012.6.1.64, 2-s2.0-84862922101. 22375173 PMC3286741

[172] Vemulapalli K. C. , Lahr R. E. , and Rex D. K. , Most Large Colorectal Polyps Missed by Gastroenterology Fellows at Colonoscopy are Sessile Serrated Lesions, Endoscopy International Open. (2022) 10, no. 05, E659–e63, 10.1055/a-1784-0959. 35571477 PMC9106434

[173] Jain A. , Javier J. , Nguyen-Ngo K. , and Tadros M. , Colonoscopy Quality Indicators in Transition: From Adenoma Detection Rate to Serrated Lesion Detection and Beyond, Diagnostics (Basel, Switzerland). (2026) 16, no. 2, 10.3390/diagnostics16020258. PMC12839587 41594234

[174] Desai M. , Anderson J. C. , Kaminski M. et al., Sessile Serrated Lesion Detection Rates During Average Risk Screening Colonoscopy: a Systematic Review and meta-analysis of the Published Literature, Endoscopy International Open. (2021) 9, no. 04, E610–e20, 10.1055/a-1352-4095. 33869735 PMC8043815

[175] Obuch J. C. , Pigott C. M. , and Ahnen D. J. , Sessile Serrated Polyps: Detection, Eradication, and Prevention of the Evil Twin, Current Treatment Options in Gastroenterology. (2015) 13, no. 1, 156–170, 10.1007/s11938-015-0046-y. 25623474 PMC4427517

[176] Barclay R. L. , Vicari J. J. , Doughty A. S. , Johanson J. F. , and Greenlaw R. L. , Colonoscopic Withdrawal Times and Adenoma Detection During Screening Colonoscopy, New England Journal of Medicine. (2006) 355, no. 24, 2533–2541, 10.1056/nejmoa055498, 2-s2.0-33845500545. 17167136

[177] Zhao S. , Yang X. , Wang S. et al., Impact of 9-Minute Withdrawal Time on the Adenoma Detection Rate: A Multicenter Randomized Controlled Trial, Clinical Gastroenterology and Hepatology. (2022) 20, no. 2, e168–e181, 10.1016/j.cgh.2020.11.019. 33220526

